# The *Pax6* master control gene initiates spontaneous retinal development via a self-organising Turing network

**DOI:** 10.1242/dev.185827

**Published:** 2020-12-23

**Authors:** Timothy Grocott, Estefania Lozano-Velasco, Gi Fay Mok, Andrea E. Münsterberg

**Affiliations:** School of Biological Sciences, University of East Anglia, Norwich Research Park, Norwich NR4 7TJ, UK

**Keywords:** Eye development, Follistatin, Pattern formation, Pax6, Self-organisation, TGFβ

## Abstract

Understanding how complex organ systems are assembled from simple embryonic tissues is a major challenge. Across the animal kingdom a great diversity of visual organs are initiated by a ‘master control gene’ called *Pax6*, which is both necessary and sufficient for eye development. Yet precisely how *Pax6* achieves this deeply homologous function is poorly understood. Using the chick as a model organism, we show that vertebrate *Pax6* interacts with a pair of morphogen-coding genes, *Tgfb2* and *Fst*, to form a putative Turing network, which we have computationally modelled. Computer simulations suggest that this gene network is sufficient to spontaneously polarise the developing retina, establishing the first organisational axis of the eye and prefiguring its further development. Our findings reveal how retinal self-organisation may be initiated independently of the highly ordered tissue interactions that help to assemble the eye *in vivo*. These results help to explain how stem cell aggregates spontaneously self-organise into functional eye-cups *in vitro*. We anticipate these findings will help to underpin retinal organoid technology, which holds much promise as a platform for disease modelling, drug development and regenerative therapies.

## Abstract (precis)

**Highlighted Article:** Pax6 can induce ectopic eye development *in vivo*, while retinal organoids can self-organise *in vitro*. We identify a Pax6 Turing network that could explain both phenomena.

## INTRODUCTION

**Fig. 1.**
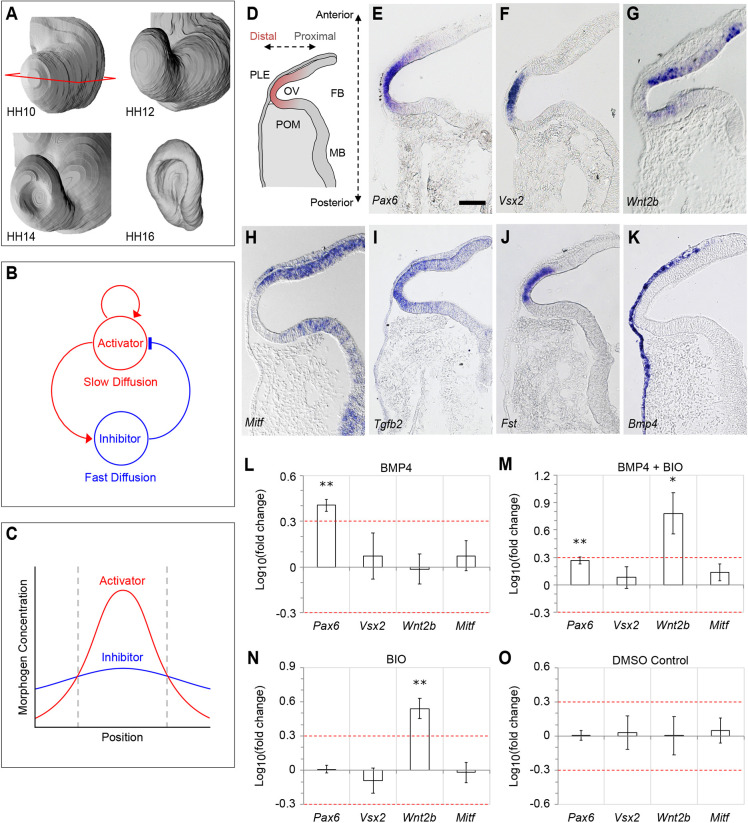
**Bmp and canonical Wnt signalling do not directly synergise to induce proximal identity in the optic vesicle.** (A) 3D surface reconstructions of the chick optic vesicle/cup from stages HH10-HH16. The horizontal plane of sectioning is indicated for stage HH10. (B,C) An activator-inhibitor type Turing network. (B) A slow-diffusing activator morphogen drives its own production and that of a faster-diffusing inhibitor morphogen, which inhibits the activator. (C) The network yields a molar excess of activator over inhibitor at their common source, but an excess of inhibitor away from their source. (D) Schematic representation of a horizontal section through the stage HH10 chick optic vesicle identifying neighbouring tissues, anterior-posterior axis and proximal-distal axis. OV, optic vesicle; PLE, presumptive lens ectoderm; POM, periocular mesenchyme; FB, forebrain; MB, midbrain. (E-K) The HH10 optic vesicle is polarised along a proximal-distal axis. Horizontal sections reveal polarised expression of the marker genes (E) *Pax6*, (F) *Vsx2*, (G) *Wnt2b*, (H) *Mitf*, (I) *Tgfb2* and (J) *Fst*. (K) *Bmp4* is expressed in the overlying presumptive lens ectoderm. (L-O) RT-QPCR analysis of proximal and distal marker gene expression following 16 h exposure to (L) Bmp4 only, (M) Bmp4 and BIO (a canonical Wnt agonist) in combination, (N) BIO only and (O) DMSO carrier control. Values plotted are Log10(mean fold change)±s.e.m. Red guidelines indicate the levels of ±2-fold change in gene expression. *P<0.05, **P<0.01 (Student's paired *t*-test). Scale bars: 150 µm.

Positional cues that govern cell fate decisions in the embryo may arise at multiple organisational levels: cell intrinsically (e.g. asymmetric cell divisions), tissue intrinsically (e.g. reaction-diffusion mechanisms), tissue extrinsically (e.g. inductive tissue interactions) or some combination of these. Historically, the early patterning of cell fates within the vertebrate eye has emphasised inductive interactions, stemming from Spemann's seminal work on lens induction (Spemann, 1901). These inductive interactions furnish positional information to coordinate self-assembly of the various tissues that make up the vertebrate camera eye, including the optic vesicle of the forebrain, which generates the retina, and the overlying presumptive lens ectoderm (Gunhaga, 2011). In the embryo, interactions with neighbouring tissues help to remodel the hemi-spherical optic vesicle into a bi-layered optic cup (Fig. 1A). Yet this vesicle-to-cup transformation is spontaneously recapitulated by stem cell-derived retinal organoids *in vitro* (Eiraku et al., 2011), revealing that a hitherto unsuspected tissue-intrinsic mechanism suffices to self-organise the primary retinal axis. Here, we provide evidence for a self-organising mechanism centred on the transcription factor-coding gene paired box 6 (*Pax6*).

*Pax6* has been called an eye master control gene (Gehring, 1996) and is necessary for eye development across much of the animal kingdom, from flies to humans (Hill et al., 1991; Hodgson and Saunders, 1980; Hoge, 1915; Nakayama et al., 2015). Mis-expression of mammalian or cephalopod *Pax6* genes triggers the spontaneous development of ectopic compound eyes in arthropods (Halder et al., 1995; Tomarev et al., 1997), as well as supernumerary camera eyes in vertebrates (Chow et al., 1999). This deeply homologous function, whereby a shared *Pax6* genetic apparatus builds eye structures that are morphologically and phylogenetically distinct (Shubin et al., 1997), is poorly understood.

The transforming growth factor β (Tgfβ) signalling pathway (Massagué, 1998) is transduced by ligand dimers that assemble hetero-tetrameric receptor complexes. The activated receptor complex then phosphorylates Smad2 and Smad3 proteins, which assemble with Smad4 before translocating to the nucleus where they interact with transcription factors to regulate gene expression. Whereas Smad2, Smad3 and Smad4 transduce Tgfβ, activin and Nodal signals, an inhibitory Smad7 antagonises this pathway cell-autonomously. Additionally, secreted antagonists, such as follistatin (Fst) act non-cell-autonomously by blocking ligand-receptor interactions (Iemura et al., 1998; Nakamura et al., 1990; Nogai et al., 2008). Smad4 is shared with the parallel bone morphogenetic protein (Bmp) signalling pathway, the signals of which are transduced by Smad1, Smad5 and Smad8, and inhibited by Smad6. We have previously reported that Pax6 protein function, and thus autoregulation, is inhibited via a direct Tgfβ-dependent interaction with Smad3, which inhibits Pax6-DNA binding (Grocott et al., 2007). Subsequently, we showed that Tgfβ signals emanating from the peri-ocular neural crest mesenchyme suppress Pax6 to align the lens with the optic vesicle (Grocott et al., 2011).

The molecular mechanisms by which tissues spontaneously generate patterns was first considered by Turing, who coined the term ‘morphogen’ to describe such molecules and devised reaction-diffusion models to simulate them (Turing, 1952). Gierer and Meinhardt later independently conceived of their activator-inhibitor model – a Turing network in which a slow-diffusing activator morphogen drives both its own production and that of a faster diffusing inhibitor morphogen, which suppresses the activator (Fig. 1B) (Gierer and Meinhardt, 1972). Thus, there arises a molar excess of activator over inhibitor at their source, where positive-feedback dominates, but a molar excess of inhibitor away from their source, where negative-feedback dominates (Fig. 1C).

Here, we describe a putative self-organising Turing network (Turing, 1952) comprising *Pax6* and a pair of morphogen-coding genes: transforming growth factor β2 (*Tgfb2*) and follistatin (*Fst*). Using reaction-diffusion modelling we show how this gene network may spontaneously polarise the optic vesicle to trigger self-organisation of the vertebrate retina.

## RESULTS

### Extrinsic Bmp signals drive *Pax6* expression in the distal optic vesicle

Optic vesicle polarisation is apparent from Hamburger and Hamilton (1992) stage HH10 in the chick, evidenced by differential gene expression along a proximal-distal axis (Fig. 1D): *Pax6* and visual system homeobox 2 (*Vsx2*; formerly *Chx10*) are expressed distally (Fig. 1E,F), whereas microphthalmia-associated transcription factor (*Mitf*) and Wnt family member 2b (*Wnt2b*; formerly *Wnt13*) are expressed proximally (Fig. 1G,H). We additionally report that two further genes, transforming growth factor β2 (*Tgfb2*) and follistatin (*Fst*) are co-expressed with *Pax6* in the distal optic vesicle (Fig. 1I,J). Neither *Tgfb2* nor *Fst* expression is detected in the overlying presumptive lens ectoderm.

As the optic vesicles evaginate between stages HH8 and HH10, they encounter bone morphogenetic protein (Bmp) family growth factors from the overlying surface ectoderm (e.g. *Bmp4*; Fig. 1K). Bmps are implicated in establishing both distal and proximal cell identities within the optic vesicle; Bmp alone promotes distal character (Pandit et al., 2015), whereas combined with canonical Wnt signalling it was proposed to induce proximal character (Steinfeld et al., 2013). Consistently, we found that exposing HH10 optic vesicle explants to Bmp4 ligand for 16 h *in vitro* led to an upregulation of distal *Pax6* (2.35±0.19 fold, mean±standard deviation; *P*<0.01; *n*=4) as measured by RT-QPCR (Fig. 1L). The remaining distal (*Vsx2*) and proximal (*Wnt2b* and *Mitf*) markers were not significantly affected (Fig. 1L). Following combined exposure to both Bmp4 and the Wnt agonist BIO (6-bromoindirubin-3′-oxime; GSK3 inhibitor) (Meijer et al., 2003), *Pax6* (1.88±0.38 fold; *P*<0.05; *n=*5) was similarly affected (Fig. 1M), while the proximal marker *Wnt2b* was additionally upregulated (9.28±7.89 fold; *P*<0.05; *n=*5), suggesting that *Wnt2b* may auto-regulate. Wnt activation alone induced proximal *Wnt2b* (3.69±1.43 fold; *P*<0.01; *n=*4) without significantly affecting distal markers (Fig. 1N), while exposure to DMSO (carrier for BIO) had no impact (Fig. 1O). These data do not support a direct synergism between Bmp and Wnt signalling in establishing proximal-distal polarity, as their combined action is merely additive.

**Fig. 2.**
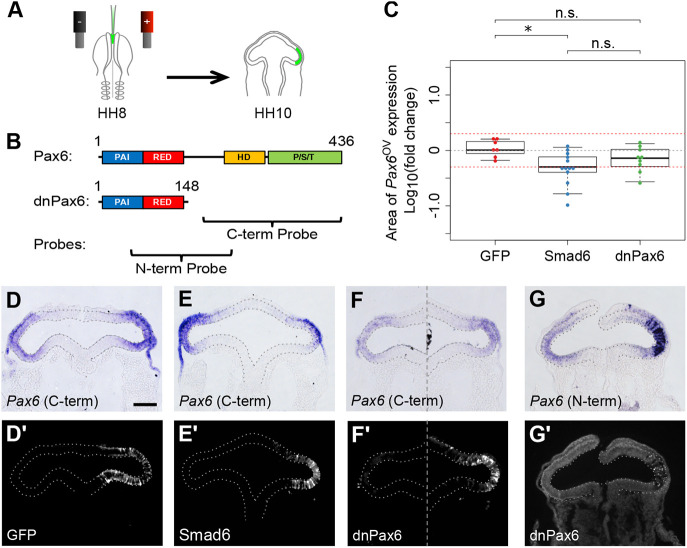
**Bmp signalling is required for *Pax6* gene expression in the distal optic vesicle.** (A) DNA expression constructs were injected into the lumen of the anterior neural tube of stage HH8 chick embryos and electroporated to transfect a single prospective optic vesicle, the other serving as an untransfected internal control. Embryos were cultured for 10-12 h overnight until stage HH10 when they were analysed. (B) Schematic showing the domain structure of the major Pax6 isoform compared with the truncated dominant-negative Pax6 (dnPax6). PAI and RED, DNA-binding subdomains that make up the N-terminal paired domain; HD, DNA-binding homeodomain; P/S/T, C-terminal proline/serine/threonine-rich transactivation domain. Antisense RNA probes against C- or N-terminal sequences respectively detect endogenous Pax6 transcripts only or endogenous Pax6 and dnPax6 together. (C) The sectional area of *Pax6* gene expression was measured and compared between electroporated and non-electroporated optic vesicles for each embryo. Log10(fold change) was plotted for embryos electroporated with GFP control construct, Smad6+GFP construct or dnPax6+GFP construct. Red guidelines indicate the level of ±2-fold change in sectional expression area. *P<0.05; n.s. indicates *P*≥0.05 (one-way ANOVA with Tukey's post-hoc test). (D-G) Endogenous *Pax6* gene expression following transfection with (D) GFP control, (E) Smad6+GFP and (F,G) dnPax6+GFP, and (D′-G′) anti-GFP immunofluorescence showing the location of (D′) GFP transfected cells, (E′) Smad6+GFP transfected cells and (F′,G′) dnPax6+GFP transfected cells. Scale bars: 100 µm. Immunofluorescence in G′ is heavily quenched by strong *in situ* staining. Optic vesicles are indicated by broken outlines.

To validate the interaction between Bmp signalling and *Pax6* expression *in vivo*, we performed electroporation-mediated gene transfer to mis-express the cell-autonomous Bmp inhibitor *Smad6* in single optic vesicles, while unelectroporated contralateral vesicles served as internal negative controls (Fig. 2A). In comparison with mis-expression of a benign enhanced green fluorescent protein (GFP; 1.13±0.37 fold; *n*=7; Fig. 2C,D), *Smad6* caused an asymmetric reduction in the area of *Pax6* expression between transfected and contralateral control vesicles (0.56±0.31 fold; *P*<0.05; *n*=13; Fig. 2C,E). This confirms that distal *Pax6* expression *in vivo* requires upstream Bmp.

Auto-regulation of *Pax6* has been reported in a number of tissues, including the lens (Ashery-Padan et al., 2000). To test for *Pax6* auto-regulation in the optic vesicle, a C-terminally truncated dominant-negative *Pax6* gene (*dnPax6*) (Grocott et al., 2007) was mis-expressed unilaterally, while a C-terminal riboprobe was used to selectively detect endogenous *Pax6* expression (Fig. 2B). *dnPax6* did not disrupt endogenous *Pax6* expression (0.75±0.36 fold; *P*>0.05; *n*=9; Fig. 2C,F) compared with the GFP control, nor could we distinguish a difference between *dnPax6* and *Smad6* mis-expression (Fig. 2C; *P*>0.05). To confirm that *dnPax6* was overexpressed relative to endogenous *Pax6*, an N-terminal riboprobe was used to collectively detect both endogenous *Pax6* and exogenous *dnPax6* expression (Fig. 2G). Thus, although distal *Pax6* expression in the optic vesicle requires Bmp signalling *in vivo*, we cannot exclude the possibility that upstream Bmp action may mask subsequent *Pax6* auto-regulation.

### *Pax6* drives expression of *Tgfb2* and its antagonist *Fst* in the distal optic vesicle

**Fig. 3.**
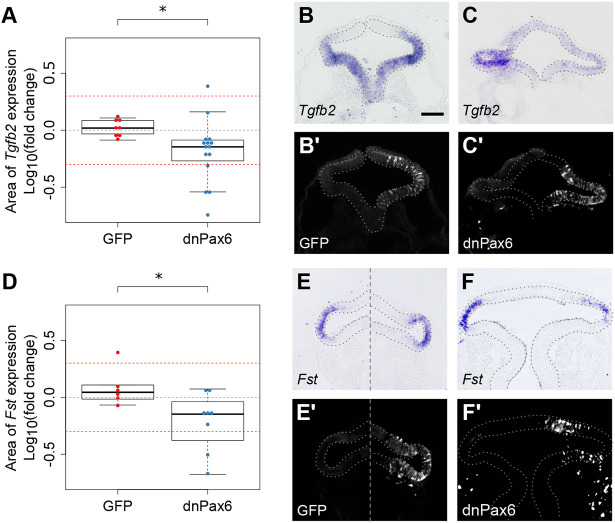
**Pax6 function is required for expression of *Tgfb2* and *Fst*.** (A-C′) *Tgfb2* gene expression was assessed 12 h after electroporation of GFP or dnPax6+GFP into a single optic vesicle. (A) Sectional areas of *Tgfb2* gene expression were measured and compared between electroporated and non-electroporated optic vesicles for each embryo. Log10(fold change) was plotted for each embryo. Red guidelines indicate the level of ±2-fold change in sectional expression area. (B,C) *Tgfb2* gene expression following electroporation with (B) GFP control and (C) dnPax6+GFP, and (B′,C′) anti-GFP immunofluorescence showing the location of (B′) GFP transfected cells and (C′) dnPax6+GFP transfected cells. (D-F) *Fst* expression was assessed 12 h after electroporation with GFP or dnPax6+GFP. (D) Sectional areas of *Fst* gene expression were measured and compared between electroporated and non-electroporated optic vesicles for each embryo. Log10(fold change) was plotted for each embryo. Red guidelines indicate the level of ±2-fold change in sectional expression area. (E,F) *Fst* gene expression following electroporation with (E) GFP control and (F) dnPax6+GFP, and (E′,F′) anti-GFP immunostaining showing the location of (E′) GFP transfected cells and (F′) dnPax6+GFP transfected cells. Optic vesicles are indicated by broken outlines. *P<0.05 (Welch's two-sample *t*-test). Scale bars: 100 µm.

Migratory neural crest cells reach the optic vesicle at stage HH10, where they contribute to the periocular mesenchyme and are thought to induce proximal and suppress distal gene expression via Tgfβ subfamily signalling (Fuhrmann et al., 2000; Grocott et al., 2011). Exogenously supplied Tgfβ subfamily ligand (activin A) was reported to induce proximal (*Wnt2b* and *Mitf*) and inhibit distal (*Pax6* and *Vsx2*) gene expression in explant cultures (Fuhrmann et al., 2000). In contrast to this tissue-extrinsic induction mechanism, stem cell-derived retinal organoids are reported to polarise tissue-autonomously, exemplified by the spontaneous acquisition of proximal Wnt activity (Hasegawa et al., 2016). This raises the possibility of a redundant tissue-intrinsic polarising activity. Given that distal *Tgfb2* expression correlates with *Pax6* (Fig. 1E,I) we asked whether *Pax6* might induce *Tgfb2* to activate proximal target genes tissue-autonomously. In comparison with GFP controls (1.06±0.17 fold; *n*=8; Fig. 3A,B), mis-expression of *dnPax6* in single optic vesicles diminished *Tgfb2* expression relative to contralateral control vesicles (0.79±0.54 fold; *P*<0.05; *n*=15; Fig. 3A,C). Thus, the *Pax6* master controller is required for *Tgfb2* expression in the distal vesicle, consistent with a report of Pax6-binding sites located within the *Tgfb2* promoter (Wolf et al., 2009).

This presents a paradox, however: *Tgfb2* expression (Fig. 1I) negatively correlates with its positive targets *Wnt2b* and *Mitf* (Fig. 1G,H), yet positively correlates with its negative targets *Pax6* and *Vsx2* (Fig. 1E,F) (Fuhrmann et al., 2000). How might Tgfβ pathway activation become inverted relative to *Tgfb2* gene expression? We considered whether *Pax6* might also activate *Fst* (Fig. 1J), a Tgfβ antagonist, to grant distal immunity from Tgfβ signalling. Compared with GFP controls (1.31±0.63 fold; *n*=6; Fig. 3D,E), mis-expression of *dnPax6* in a single optic vesicle significantly reduced *Fst* expression (0.69±0.34 fold; *P*<0.05; *n*=8; Fig. 3D,F). Thus, *Pax6* function is additionally required for *Fst* expression in the distal vesicle.

The paradoxical out-of-phase expression of distal *Tgfb2* and its proximal (positive) targets might then be explained by differential diffusion of *Tgfb2* and *Fst* gene products resulting in: (1) Tgfβ2 being locally sequestered by slow-diffusing Fst within the distal vesicle, thereby preserving distal character; and (2) fast-diffusing Tgfb2 dispersing proximally away from Fst, to induce proximal character within the neighbouring proximal vesicle.

**Fig. 4.**
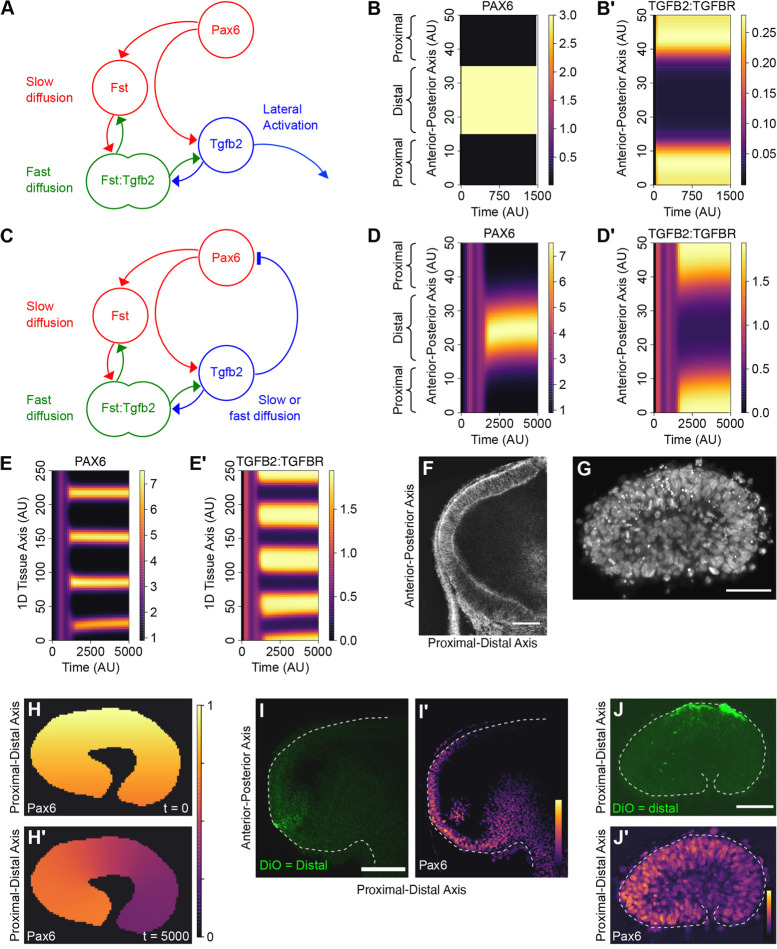
**Reaction-diffusion modelling of the *Pax6*/*Fst*/*Tgfb2* gene network.** (A) Summary of Model A, in which Pax6 drives expression of both *Fst* and *Tgfb2*, whereas Fst inhibits Tgfβ2 function via sequestration. Slow diffusion of Fst was postulated to result in local inhibition of Tgfβ2 at the source of *Pax6*/*Tgfb2*/*Fst* expression. Conversely, fast diffusion of Tgfβ2 was postulated to drive lateral activation of its downstream signalling pathway away from the *Pax6*/*Fst*/*Tgfb2*-expressing region. (B,B′) 1D numerical simulation of Model A, in which Pax6 expression is regionally restricted throughout. For all simulations, units of space, time and molecular concentrations are arbitrary. The vertical *y*-axis represents the anterior-posterior axis of the hemispherical optic vesicle, which is divided into anterior-proximal, distal and posterior-proximal domains. The plots depict the time evolution (*x*-axis) for 1D spatial distributions (*y*-axis) of (B) Pax6 and (B′) the activated Tgfβ2:Tgfβ-receptor signalling complex. (C) Summary of Model B in which Fst:Tgfβ2 complex quickly diffuses and dissociates, while Tgfβ2 additionally inhibits Pax6 transcriptional activator function. (D,D′) 1D numerical simulation of Model B in which Pax6 expression is initially homogenous but noisy. The plots depict spontaneous generation of (D) a Pax6+ ‘distal pole’ flanked by (D′) Tgfβ2:Tgfβ-receptor+ ‘proximal poles’. (E,E′) 1D numerical simulation of Model B with a larger tissue size resulting in (E) multiple Pax6+ ‘distal poles’ interspersed with (E′) Tgfβ2:Tgfβ-receptor+ ‘proximal poles’. (F) Confocal section of an HH10 tg(membrane-GFP) embryo showing optic vesicle size prior to explant culture. (G) Confocal section of a fixed optic vesicle explant showing the collapsed tissue following 16 h culture. Cell nuclei are stained with DAPI. (H,H′) 2D numerical simulation of Model B within an explant-shaped domain (Model C). (H) The initial distal-high to proximal-low Pax6 pattern is (H′) dynamically re-polarised along the longest axis of the explant. (I,I′) Partially dissected optic vesicle in which the distal end was fluorescently labelled with (I) DiO, corresponding to (I′) the Pax6+ pole revealed by immunofluorescent staining. (J,J′) Explant experiment in which (J) the distal pole of the optic vesicle was labelled with DiO during dissection. (J′) Following overnight culture, Pax6 expression has re-polarised relative to the former proximal-distal axis. Scale bars: 50 µm in F,G,J; 100 µm in I.

**Fig. 5.**
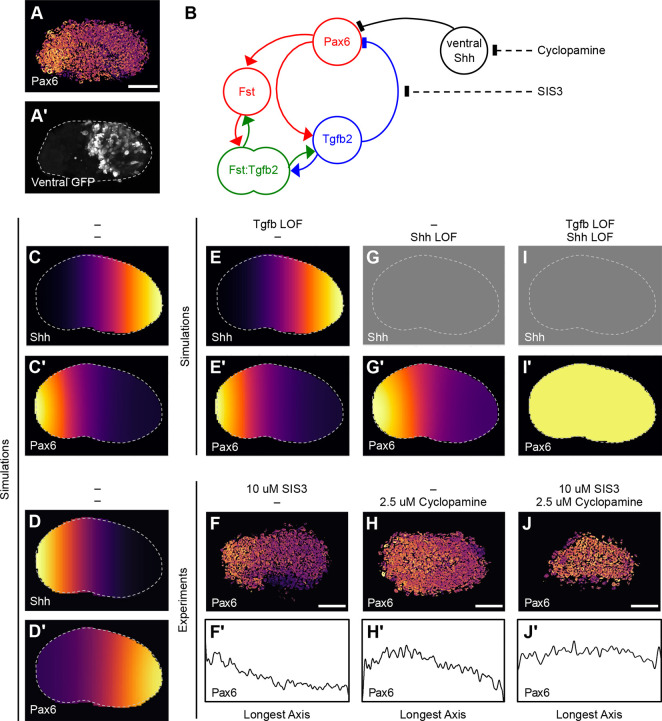
**Shh positional information and Tgf**β**-mediated self-organisation position the Pax6+ pole in cultured explants.** (A,A′) The Pax6+ pole re-aligns with the dorsal-ventral axis in explanted optic vesicles. Maximal projections of (A) Pax6 immunofluorescence normalised to DAPI and (A′) ventrally targeted GFP in a whole-mount explant. (B) Summary of Model D in which a ventral-high to dorsal-low Shh gradient inhibits Pax6 expression. The pharmacological compounds used in functional experiments are also indicated (broken lines). (C,C′) 2D numerical simulation of Model D showing (C) the ventral-high Shh gradient (C′) Pax6 re-polarisation. (D,D′) 2D numerical simulation of Model D showing (D) reversal of the Shh gradient and (D′) corresponding reversal of Pax6 polarity. (E,E′) 2D numerical simulation of Model D with Tgfβ loss of function (Tgfb LOF) showing (E) the ventral high Shh gradient and (E′) the resulting Pax6 distribution. (F,F′) Optic vesicle explants were cultured with 10 μM SIS3 for 16 h. (F) Maximum projection of Pax6 immunofluorescence normalised to DAPI. (F′) 1D profile plot of Pax6 abundance along the longest (horizontal) axis of the explant. (G,G′) 2D numerical simulation of Model D with Shh loss of function (Shh LOF) showing (G) absence of Shh positional information and (G′) the resulting Pax6 distribution. (H,H′) Optic vesicle explants were cultured with 2.5 μM cyclopamine for 16 h. (H) Maximum projection of Pax6 immunofluorescence normalised to DAPI. (H′) 1D profile plot of Pax6 abundance along the longest (horizontal) axis of the explant. (I,I′) 2D numerical simulation of Model D with both Tgfβ loss of function and Shh loss of function showing (I) absence of Shh positional information and (I′) the resulting Pax6 distribution. (J,J′) Optic vesicle explants were cultured with both 10 μM SIS3 and 2.5 μM cyclopamine for 16 h. (J) Maximum projection of Pax6 immunofluorescence normalised to DAPI. (J′) 1D profile plot of Pax6 abundance along the longest (horizontal) axis of the explant. Scale bars: 50 μm.

To test whether this hypothesis is plausible, we examined a reaction-diffusion model of the interactions summarised in Fig. 4A (Model A, Fig. S1; see supplementary information) and performed numerical simulations in one dimension only to represent the anterior-posterior axis (comprising anterior-proximal, distal and posterior-proximal domains) of the optic vesicle. Simulations were performed with both zero-flux (Fig. 5) and periodic (Movies 1 and 2) boundary conditions to represent dissected optic vesicle explants and spherical organoids, respectively.

A variety of diffusion ratios for Tgfb2 dimers and Fst monomers versus Fst:Tgfb2 complexes were explored (e.g. Fig. 4B,B′; Fig. S2; Movie 1). Simulations demonstrated that local inhibition and lateral-activation of Tgfb signalling may occur if the diffusion rate of Fst:Tgfb2 complexes exceeds that of Fst monomers. Although initially counter-intuitive, there is precedent for ligand:antagonist complexes that disperse faster than their individual constituents (Esteve et al., 2011), and our subsequent simulations assume this condition is satisfied.

### *Pax6*, *Fst* and *Tgfb2* form a self-organising Turing network that can dynamically polarise the optic vesicle

Given that Tgfb signalling is known to disrupt Pax6 protein function (Grocott et al., 2007), such local inhibition and lateral activation of Tgfβ signalling equate to local positive feedback and lateral-inhibition of the *Pax6* master control gene, respectively (Fig. 4C). This is functionally equivalent to a simple activator-inhibitor type (Fig. 1B) Turing network (Gierer and Meinhardt, 1972; Turing, 1952), which can serve as a spontaneous pattern generator: *Pax6* and *Fst* provide a short-range auto-regulating activator; and *Tgfb2* is the long-range inhibitor (compare Fig. 1B with Fig. 4C). To explore whether the network of Fig. 4C possesses spontaneous polarising activity, we simply extended Model A to include inhibition of Pax6 function by Tgfβ signalling (Model B; Fig. S3; see supplementary information). Simulations showed that an initially homogenous but noisy *Pax6* distribution is readily converted into a polarised pattern, wherein *Pax6* expression becomes regionally restricted (Fig. 4D) and out-of-phase with Tgfb receptor activation (Fig. 4D′; Fig. S4; Movie 2). Additionally, simulating larger tissue sizes results not in a larger *Pax6*-expressing distal pole, but in a greater number of *Pax6*-expressing distal poles of approximately equal size (Fig. 4E,E′). This hallmark feature of Turing networks is remarkably consistent with observations of retinal organoid cultures in which stem cell aggregates yielded between one and four retinas each (Eiraku et al., 2011).

Similarly, reducing tissue size limits the number rather than the size of pattern elements generated by a Turing network so that, for example, a single ‘spot’, half a ‘spot’ (i.e. a gradient) or no ‘spot’ is generated. When cultured as isolated explants in the absence of serum, polarised HH10 optic vesicles (e.g. Fig. 4F) collapse into compact spheroids (Fig. 4G), reducing the longest dimension of this tissue to ≤0.5 fold. To better understand how the *Pax6*/*Fst*/*Tgfb2* network might respond in this situation, we performed 2D simulations of Model B on an explant-shaped domain with an initial distal-high to proximal-low Pax6 pattern (Model C; Fig. 4H; see supplementary information). For these simulations, we explored both zero-flux and fixed-boundary conditions, disregarding the latter as the former agreed more closely with experimental observations. It may be interpreted that adsorption of morphogens to extracellular matrix and cell-surface proteins within explants prevents a significant outward flux, while the absence of morphogens from the defined bathing medium prevents an inward flux.

Owing to the reduced tissue size, this proximal-distal pattern proved unstable and Pax6 expression quickly re-polarised to form a gradient along the longest axis of the explant (i.e. perpendicular to the former proximal-distal axis; Fig. 4H′; Fig. S5; Fig. S6; Movie 3). To test this model prediction, optic vesicles were dissected for explant culture, during which their distal poles were labelled with DiO (Fig. 4I). Immunostaining of a partially dissected optic vesicle verifies that DiO labelling coincides with the initial Pax6^+^ distal pole (Fig. 4I′). Following overnight culture, however, Pax6 expression no longer coincides with the distal DiO label but instead re-polarises along the longest axis of each explant (Fig. 4J,J′) consistent with simulations. This suggests that the *Pax6*/*Fst*/*Tgfb2* network can dynamically repolarise its expression in a self-organising fashion.

### Intrinsic positional information constrains *Pax6*/*Fst*/*Tgfb2* self-organisation

In explant culture, optic vesicles are isolated from inductive tissue interactions and thus from extrinsic positional information. However, we questioned whether Pax6 repolarisation might be influenced by intrinsic positional information. A ventral-high to dorsal-low gradient of Sonic hedgehog (Shh) signalling activity exists within the optic vesicle, which is known to restrict the ventral extent of *Pax6* expression (Ekker et al., 1995; Macdonald et al., 1995). Might Shh positional information push the Pax6^+^ pole towards the dorsal side of the explant? Electroporation of a GFP expression construct was targeted to the ventral optic vesicle prior to dissection and overnight explant culture. Whole-mount immunofluorescence staining showed that the Pax6^+^ pole negatively correlates with ventral GFP expression (100% of explants; *n*=5; Fig. 5A,A′), supporting this idea.

To explore how Shh positional information might interact with the *Pax6*/*Fst*/*Tgfb* network, we extended Model C by incorporating Shh suppression of *Pax6* into the governing equations (Model D; Fig. 5B; see supplementary information) while adding a Shh positional information gradient (Fig. 5C; Fig. S7). Simulations showed that the Pax6^+^ pole reorientates away from the ventral-high end of the Shh gradient (Fig. 5C′; Fig. S8; Fig. S9) as was observed experimentally (Fig. 5A,A′). Moreover, inverting the Shh gradient (Fig. 5D) caused a reversal of *Pax6* polarity (Fig. 5D′).

Exploring Model D, we next simulated the ability of *Pax6* to repolarise in the absence of Tgfβ-mediated self-organisation and found that the Shh positional information gradient was sufficient to generate a dorsal Pax6^+^ pole (Fig. 5E,E′). This prediction was tested experimentally by culturing optic vesicle explants in the presence of a Smad3 inhibitor, SIS3 (Jinnin et al., 2006). As Tgfβ inhibits Pax6 protein function via its specific and direct interaction with Smad3 (Grocott et al., 2007), SIS3 should block the inhibition of Pax6 by Tgfβ2 (Fig. 5B). Following overnight culture with 10 μM SIS3, optic vesicle explants still exhibited distinct Pax6*^+^* poles (91% of explants; *n*=11; Fig. 5F,F′) as predicted. These data show that Tgfβ-mediated self-organisation is not required for *Pax6* polarisation in cultured explants, presumably due to the redundant action of Shh positional information.

Model D simulations lacking Shh positional information (Shh LOF; Fig. 5G) predicted that the *Pax6*/*Fst*/*Tgfb2* network should suffice to generate a Pax6^+^ pole in the absence of Shh activity (Fig. 5G′). To test this, optic vesicle explants were cultured overnight with 2.5 μM cyclopamine: a steroidal alkaloid that inhibits the Hedgehog pathway transducer Smoothened (Chen et al., 2002). As predicted, explants still exhibited Pax6^+^ poles in the absence of Shh activity (82% of explants; *n*=11; Fig. 5H,H′). Thus, Shh positional information is not required for *Pax6* polarisation in optic vesicle explants, suggesting that the *Pax6*/*Fst*/*Tgfb2* network is sufficient to self-organise the Pax6^+^ pole. However, although still polarised in the absence of Shh positional information, *Pax6* expression is subtly upregulated both in simulations (compare Fig. 5E′ with 5G′) and in experiments (compare Fig. 5F with 5H).

Further Model D simulations predicted that simultaneous loss of both Shh positional information (Fig. 5I) and Tgfβ-mediated self-organisation should prevent *Pax6* polarisation in cultured explants (Fig. 5I′); instead of polarising, Pax6 was expressed uniformly throughout the simulated explant. Consistent with this, optic vesicle explants cultured with both 2.5 μM cyclopamine and 10 μM SIS3 mostly failed to exhibit *Pax6* polarisation as expression was approximately uniform across their lengths (67% of explants; *n*=12; Fig. 5J,J′). In other words, the *Pax6*/*Fst*/*Tgfb2* network appears to be both sufficient (Fig. 5H,H′) and necessary (Fig. 5J,J′) to self-organise *Pax6* polarisation in the absence of positional information.

### The *Pax6*/*Fst*/*Tgfb2* gene network regulates distal neural retinal identity *in vivo*

The preceding data suggest that, although the *Pax6*/*Fst*/*Tgfb2* network may freely self-organise in isolation (e.g. in retinal organoids), *in vivo* this network is constrained by intrinsic (e.g. Shh) and extrinsic (e.g. Bmp4) positional information to ensure correct alignment of the distal Pax6^+^ pole within the camera eye. Thus, functional perturbations *in vivo* are not expected to drive the kind of dynamic re-polarisation observed in cultured explants. How then might functional perturbation of the *Pax6*/*Fst*/*Tgfb2* network impact optic vesicle patterning *in vivo*?

**Fig. 6.**
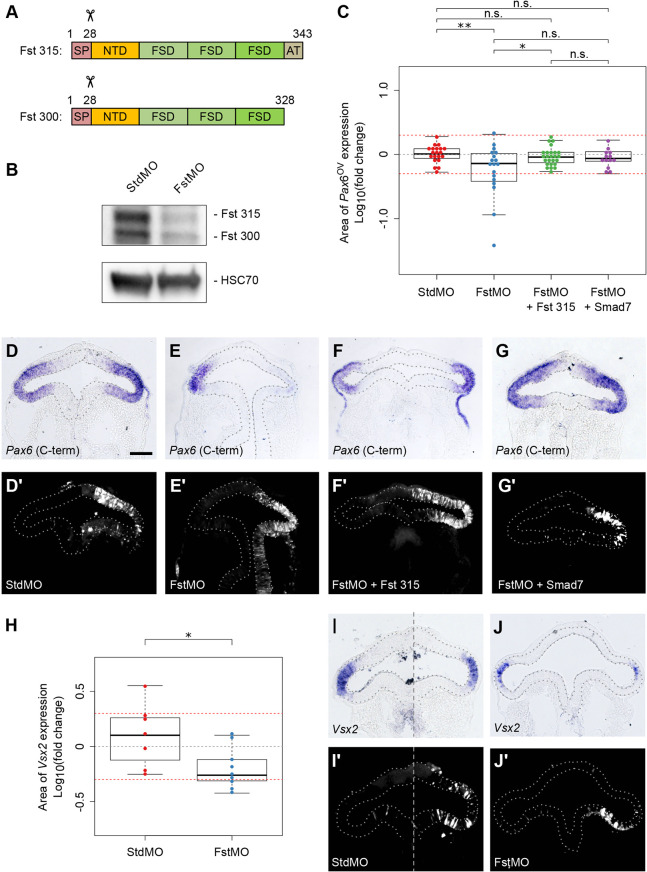
***Fst* gene function is required for correct optic vesicle polarisation via distal inhibition of Tgfβ signalling.** (A) Schematic showing domain structures encoded by naturally occurring Fst transcripts. The shorter Fst 300 is generated by alternative splicing. SP, 28 amino acid signal peptide cleaved co-translationally; NTD, N-terminal domain; FSD, follistatin domain; AT, acidic tail. (B) Western blot validation of Fst 315 and Fst 300 protein knockdown by FstMO but not by StdMO in cultured chick embryo cells. (C-G′) Sectional area of *Pax6* gene expression was assessed 12 h after co-electroporation of single optic vesicles with control/experimental morpholinos plus various gene expression constructs. (C) Sectional area of *Pax6* gene expression was measured and compared between electroporated and non-electroporated optic vesicles for each embryo. Log10(fold change) was plotted for each embryo. Red guidelines indicate the level of ±2-fold change in sectional expression area. (D-G) *Pax6* gene expression following co-electroporation of (D) standard control morpholino (StdMO)+GFP, (E) *Fst* morpholino (FstMO)+GFP, (F) FstMO+Fst gene expression construct and (G) FstMO+Smad7 gene expression construct. (D′-G′) FITC-labelled (D′) StdMO fluorescence, (E′) FstMO fluorescence, (F′) FstMO fluorescence and (G′), FstMO fluorescence, showing the location of transfected cells. (H-J′) Sectional area of *Vsx2* gene expression was assessed 12 h after co-electroporation of single optic vesicles with control/experimental morpholino. (H) Sectional area of *Vsx2* gene expression was measured and compared between electroporated and non-electroporated optic vesicles for each embryo. Log10(fold change) was plotted for each embryo. Red guidelines indicate the level of ±2-fold change in sectional expression area. (I,J) *Vsx2* gene expression following co-electroporation of (I) StdMO+GFP and (J) FstMO+GFP, and (I′,J′) FITC-labelled (I′) StdMO fluorescence and (J′) FstMO fluorescence showing the location of transfected cells. Optic vesicles are indicated by broken outlines. *P<0.05; **P<0.01 (C, one-way ANOVA with Tukey's post-hoc test; H, Welch's two-sample *t*-test). Scale bars: 100 µm.

According to our model, interference with *Fst* gene expression should de-repress Tgfβ signalling and inhibit Pax6 protein function in the distal vesicle, via the direct Tgfβ-dependent interaction of Smad3 with Pax6 (Grocott et al., 2007). Moreover, if Pax6 auto-regulates in the distal vesicle, this should manifest as a Tgfβ-mediated reduction in *Pax6* gene expression. To test this prediction, we employed morpholino oligonucleotides to suppress translation of Fst 315 and Fst 300 isoforms (Fig. 6A) within single optic vesicles. *Pax6* expression was then compared between these and unperturbed contralateral vesicles. Fst morpholino (FstMO) was first shown to suppress endogenous translation of both Fst isoforms in cultured chick embryonic cells via western blotting when compared with a standard control morpholino (StdMO) that does not target *Fst* (Fig. 6B).

*In vivo*, StdMO controls had no impact on *Pax6* expression in transfected optic vesicles (1.05±0.31 fold; *n*=20; Fig. 6C,D). In comparison, FstMO reduced *Pax6* expression in transfected vesicles (0.76±0.50 fold; *P*<0.01; *n*=18; Fig. 6C,E). We were able to rescue this loss of *Pax6* expression by co-transfecting FstMO together with an exogenous *Fst* transgene that evades FstMO and encodes the Fst 315 isoform (0.98±0.35 fold; *P*>0.05; *n*=25; Fig. 6C,F). This confirmed that loss of *Pax6* was not due to a morpholino off-target effect and that *Fst* gene function is required for distal *Pax6* expression in the optic vesicle. This is consistent with earlier reports that neural induction by way of *Fst* overexpression induces *Pax6* in *Xenopus* animal cap explants (Altmann et al., 1997).

To verify that loss of *Pax6* expression is indeed due to the predicted de-repression of Tgfβ signalling, we attempted an alternate rescue by co-transfecting FstMO together with a cell-autonomous Tgfβ/Activin/Nodal pathway inhibitor, *Smad7*. As can be seen (Fig. 6C,G), no significant loss of *Pax6* expression was observed (0.91±0.31 fold; *P*>0.05; *n*=13) when Fst translation and Tgfb signalling were simultaneously suppressed.

In addition to inducing *Pax6* (Altmann et al., 1997), overexpression of *Fst* in *Xenopus* animal cap explants was reported to induce expression of the retinal photoreceptor marker *Opsin* (Hemmati-Brivanlou et al., 1994). We therefore investigated whether *Vsx2*, a distally expressed neural retinal marker (Liu et al., 1994) (Fig. 1F), is similarly affected upon disruption of the *Pax6*/*Fst*/*Tgfb2* gene network. In comparison with StdMO controls (1.51±1.05 fold; *n*=7; Fig. 6H,I), FstMO significantly reduced distal *Vsx2* expression in transfected optic vesicles (0.69±0.33 fold; *P*<0.05; *n*=9; Fig. 6H,J). Thus, de-repression of endogenous Tgfβ signalling in the distal vesicle is detrimental for correct proximal-distal patterning, including specification of the neural retina. These results are consistent with our general model and support the idea that Fst and Tgfb2 morphogens positively and negatively regulate Pax6 function, respectively, in order to polarise the optic vesicle.

## DISCUSSION

The issue of the master control mechanism of *Pax6* has now been unresolved for a quarter of a century (Cvekl and Callaerts, 2017). Here, we have shown that the vertebrate *Pax6* directs expression of a pair of morphogen coding genes, *Fst* and *Tgfb2*, which modulate Pax6 function via positive and negative feedback. This *Pax6*/*Fst*/*Tgfb2* gene network topology is consistent with an activator-inhibitor type Turing network and appears to exhibit a self-organising pattern-forming ability in the absence of positional information. This spontaneous pattern-forming potential could explain both the ability of *Pax6* to trigger ectopic eye development across the animal kingdom (Chow et al., 1999; Halder et al., 1995; Tomarev et al., 1997) and the spontaneous development of self-organising optic cups from stem cell aggregates cultured *in vitro* (Eiraku et al., 2011).

### Prerequisites for retinal self-organisation

Our reaction-diffusion simulations showed that the *Pax6*/*Fst*/*Tgfb2* gene network may act as a self-organising Turing network, providing certain assumptions are satisfied. For example, we have assumed that larger Fst:Tgfb2 complexes diffuse more quickly than smaller Fst monomers. This is counter-intuitive as pure diffusion rate is a function of molecular mass. Yet there is a precedent for this phenomenon: for example, Sfrp:Wnt complexes have been observed to diffuse further than Wnt alone (Esteve et al., 2011). We postulate that Fst monomers disperse sub-diffusively due to binding interactions with extracellular matrix components and/or cell surface factors, e.g. heparin sulfate proteoglycans (Nakamura et al., 1991) or fibronectin (Maguer-Satta et al., 2006). In the context of Fst:Tgfb2 complexes, the relevant interaction surfaces may be shielded, enabling the larger complex to disperse further and faster than its constituents.

This assumed rapid dispersal of Fst:Tgfb2 complexes is only required if Tgfb2 sequestration by Fst is reversible, which is currently unknown. Low-affinity Fst:Bmp interactions are known to be reversible, whereas high-affinity Fst:Activin interactions are effectively irreversible (Iemura et al., 1998). If Fst:Tgfb2 associate irreversibly then spontaneous pattern formation is still possible, but it changes assumptions regarding effective diffusion rates: Fst:Tgfb2 diffusion would then become irrelevant and, instead, Tgfb2 dimers must diffuse faster than Fst monomers (Murray, 2003).

### Self-organisation versus positional information *in vivo*

**Fig. 7.**
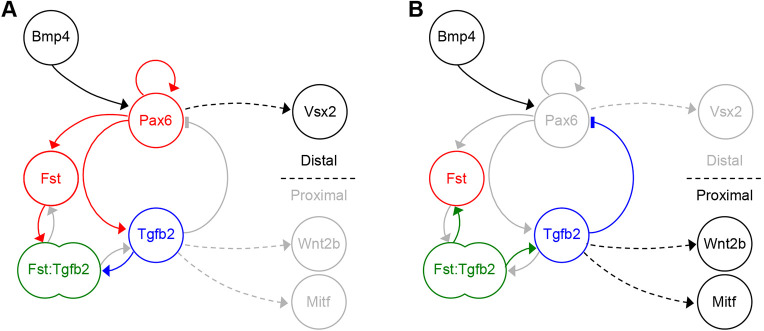
**Proposed *Pax6*/*Fst*/*Tgfb2* network function during optic vesicle polarisation *in vivo*.** (A) At the prospective distal pole, *Pax6* expression is promoted by upstream Bmp and reinforced via auto-regulation. Pax6 drives distal expression of *Fst*, *Tgfb2* and downstream *Vsx2*. A molar excess of slow-diffusing Fst over Tgfβ receptors is postulated to reversibly sequester Tgfβ2 into fast-diffusing Fst:Tgfβ2 complexes. (B) At the prospective proximal vesicle, dissociation of fast-diffusing Fst:Tgfβ2 complexes is postulated to release Tgfβ2. A molar excess of Tgfβ receptors over slow-diffusing Fst then permits receptor activation by Tgfβ2, causing functional inhibition of Pax6 and induction of proximal markers *Wnt2b* and *Mitf*. Interactions indicated by broken lines may be indirect.

By demonstrating how *Pax6* may drive self-organisation of the primary retinal axis, our findings offer the first mechanistic explanation of the long-known but poorly understood master control function of *Pax6*. In the embryo, we propose that this putative Turing network acts to self-organise the proximal-distal axis of the optic vesicle (as summarised in Fig. 7A,B), in concert with positional information (e.g. from previously identified inductive interactions) to ensure correct alignment with neighbouring tissues.

In Model D, we accounted for intrinsic positional information by incorporating direct suppression of *Pax6* expression by a ventral-high to dorsal-low gradient of Shh activity (Fig. 5; see supplementary information) (Ekker et al., 1995; Macdonald et al., 1995). This is a convenient abstraction, however; at later stages, the ventral extent of *Pax6* expression *in vivo* is refined via reciprocal inhibition between distal *Pax6* (prospective neural retina) and ventral *Pax2* (prospective optic stalk) (Schwarz et al., 2000), the expression of which is activated by ventral Shh (Ekker et al., 1995; Macdonald et al., 1995).

Regarding extrinsic positional information, Bmp signals from the overlying head ectoderm appear to activate the *Pax6*/*Fst*/*Tgfb2* network and may also bias proximal-distal polarity to align the distal Pax6^+^ pole with the prospective lens. This would explain why Bmps from the head ectoderm have been attributed with inducing both proximal retinal pigment epithelium (Müller et al., 2007) and distal neural retina (Pandit et al., 2015) within the optic vesicle.

We did not investigate the role of Wnt in establishing proximal identity within the optic vesicle, except to test for direct synergism between Wnt and Bmp, as previously proposed (Steinfeld et al., 2013). In the absence of such synergism, we suggest that Wnt acts downstream of the *Pax6*/*Fst*/*Tgfb2* gene network, as: (1) *Wnt2b* is a Tgfβ target gene (Fuhrmann et al., 2000) restricted to the proximal optic vesicle (Fig. 1G); and (2) expression of *Wnt2b* is absent from the peri-ocular surface ectoderm until stage HH11 (Grocott et al., 2011), prior to which polarised *Wnt2b* expression is already established within the optic vesicle itself (Fig. 1G).

In addition to the loss of inductive signals, ablation of the overlying lens ectoderm (Steinfeld et al., 2013) may permit periocular Tgfβ proteins from the surrounding neural crest mesenchyme (Fuhrmann et al., 2000; Grocott et al., 2011) to overwhelm the autonomous polarising activity of the *Pax6*/*Fst*/*Tgfb2* network. In turn, it has not escaped our attention that distal *Fst* may mediate classical lens induction (Spemann, 1901) by opposing these same lens-inhibitory Tgfb signals (Grocott et al., 2011); indeed, *Fst* overexpression induces lens crystallin expression in *Xenopus* animal cap explants (Altmann et al., 1997).

### Retinal organoids and self-organisation *in vitro*

During retinal organoid development *in vitro*, we propose that the *Pax6*/*Fst*/*Tgfb2* network may suffice to self-organise the primary axis of the retina in the absence of the well-organised positional information normally present *in vivo*. For example, ventral optic vesicle structures are absent in self-organising retinal organoids (Eiraku et al., 2011), which suggests an absence of intrinsic Shh positional information.

The comparatively chaotic nature of organoids makes them an ideal counterpart to embryonic models of development as they can unmask cryptic self-organising mechanisms and test them to breaking point; contrast the straightforward elaboration of an existing pre-pattern (Fig. 4B,B′; analogous to localised *Pax6* induction by neighbouring Bmps *in vivo*) with the more turbulent emergence of order from disorder (Fig. 4D,D′; analogous to spontaneous *Pax6* activation in retinal organoids).

In simulations of *de novo* pattern formation, the *Pax6*/*Fst*/*Tgfb2* network is observed to oscillate (Fig. 4D,D′; Movie 2). This potential for oscillation derives from the Eigenvalues associated with the Turing condition and thus from the models' governing equations and parameter choices. For example, in Model B, the tendency to oscillate may be suppressed by increasing the negative feedback that Tgfb2 exerts on Pax6. Whether or not oscillations manifest in a given simulation is further influenced by the choice of initial conditions. For example, Model B is observed to oscillate during *de novo* pattern formation (Fig. 4D,D′; Movie 2), but not when elaborating an existing pre-pattern (equivalent to the Model A simulation in Fig. 4B,B′; Movie 1). For this reason, we might expect that oscillations are more likely to arise during *de novo* pattern formation in retinal organoid cultures and less so in the embryo, where the wealth of positional information constrains the *Pax6*/*Fst*/*Tgfb2* network. Whether or not this gene network oscillates *in vitro* or *in vivo*, and the potential impact on robustness and reproducibility of organoid cultures, is yet to be investigated.

### Future directions

A future challenge will be to develop a full 3D model of optic vesicle patterning, incorporating the *Pax6*/*Fst*/*Tgfb2* Turing network together with all sources of constraining positional information. A multi-scale approach, in which the feedback between tissue patterning (via the reaction-diffusion formalism used here) and cell dynamics (e.g. via Cellular Potts, vertex or finite element approaches) could further illuminate the feedback between tissue patterning and morphogenesis. A 2D vertex model of optic cup morphogenesis has been previously reported (Eiraku et al., 2012), but a multi-scale approach will be required to fully grasp how genes determine geometry and to identify causal links between genetic and anatomical aberrations.

The identification of defined, animal-free substrates for organoid cultures is a prerequisite for clinical applications. This, and enhanced reproducibility, strongly motivate the search for alternatives to incompletely defined and animal-derived Matrigel, which has superseded laminin as the substrate of choice for *in vitro* retinogenesis (Capowski et al., 2019; Eiraku et al., 2011; Meyer et al., 2009). Interestingly, Matrigel's sixth most abundant ECM component, fibronectin (Rijal and Li, 2017), is enriched within the basement membrane of the optic vesicle *in vivo* (Krotoski et al., 1986; Kurkinen et al., 1979) and binds Fst (Maguer-Satta et al., 2006). Could ECM components such as fibronectin support self-organisation by limiting Fst diffusion relative to Tgfb2 or the Fst:Tgfb2 complex? Further studies are needed to characterise diffusion of these morphogens both *in vivo* and *in vitro*, and to clarify the role of ECM composition in supporting their differential diffusion.

Further exploration of the *Pax6*/*Fst*/*Tgfb2* network may drive future developments in retinal organoid technology and help underpin applications in disease modelling, drug discovery and regenerative therapies. Given the deeply homologous nature of the *Pax6* master control function, we would predict that *Pax6* orthologues participate in functionally homologous Turing networks in non-vertebrates, which may comprise the same or different morphogens.

## MATERIALS AND METHODS

### Chick embryos

Fertile brown hen's eggs (Henry Stewart) were incubated at 38°C in a humidified incubator until the required stage of development: HH8 for *in ovo* electroporation experiments; HH10 for *in vitro* explant experiments. The study was approved by the Animal Welfare & Ethical Review Board, School of Biological Sciences of the University of East Anglia, and all procedures were performed in accordance with the relevant guidelines and regulations.

### Explant assays

HH10 embryos were incubated with 0.25% Trypsin-EDTA at 38°C for 7 min. Trypsin was then de-activated by transferring into 20% chick serum on ice for 5 min. Embryos were then washed with Tyrode's solution and pinned onto Sylgard-coated dissection dishes. Head surface ectoderm and peri-ocular mesenchyme were carefully removed using 30 gauge syringe needles from both dorsal and ventral sides. Once cleaned, both optic vesicles were removed and held in Tyrode's solution on ice. Left and right optic vesicles were separately pooled from at least five embryos, yielding two match-paired pools for use as treated and control samples. Pooled vesicles were cultured in polyHEMA (Sigma)-coated culture wells to prevent adhesion, with DMEM-F12 media (Invitrogen) supplemented with 1× N2 (Invitrogen), 1× L-glutamate and 1× penicillin/streptomycin at 37°C and 5% CO_2_ for 16 h. Culture media for treated samples was supplemented with the following factors as required: 35 ng/ml Bmp4 (R&D Systems), 0.5 µM BIO (Sigma) with 0.1% DMSO (Sigma), 10 µM SIS3 with 0.1% DMSO (Sigma), or 2.5 µM cyclopamine (Sigma) with 0.1% 2-hydroxypropyl-β-cyclodextrin (HBC; Sigma).

### Whole-mount immunofluorescence staining of explants

Cultured explants were fixed in 4% PFA at 4°C for 90 min, dehydrated and rehydrated through methanol series. After blocking overnight at 4°C in PBTS (BSA, Triton X-100 and goat serum), explants were incubated in mouse anti-Pax6 primary antibody (diluted 1:50 in PBTS; Developmental Studies Hybridoma Bank #PAX6) for 3 days then washed in PBS-Tween. Explants were then incubated in goat anti-mouse Alexa568-conjugated secondary antibody (diluted 1:1000 in PBTS; Life Technologies A-11004) and DAPI for 3 days at 4°C, then washed in PBS-Tween. Stained explants were mounted in AF1 mounting medium (Citifluor) and *z*-stack images were generated using a Zeiss LSM980 confocal instrument. Relative quantification of nuclear Pax6 fluorescence was performed by normalising to DAPI using the Atlas Toolkit plug-in for FIJI/ImageJ (Grocott et al., 2016) as described.

### Quantitative RT-PCR

Explant samples were lysed in 1 ml Trizol (Ambion) and processed for total RNA extraction. RNA samples were digested with DNase I (Ambion) and re-extracted by acidic phenol/chloroform. RNA concentrations were determined by NanoDrop ND-1000 Spectrophotometer. For each experiment, equal quantities of treated and control sample RNA (typically between 0.1–0.6 µg) were used as a template for first strand cDNA synthesis using Superscript II reverse transcriptase (Invitrogen) and random hexamers. cDNAs were diluted 1:20 before relative quantitation of transcript levels by real-time PCR using SYBR Green master mix (Applied Biosystems) and target-specific primers (Table S1). Relative transcript quantification was via the standard curve method, and target gene expression was normalised to the reference gene β-Actin. Fold changes were calculated for each matched-pair (treated/control) then log-transformed to bring data closer to a normal distribution (verified by a Shapiro–Wilk test) prior to plotting and null hypothesis significance testing. These were plotted as mean±s.d. Student's paired *t*-test was used to calculate the probability of the observed (or more extreme) differences between match-paired (treated and control) sample means, assuming that the null hypothesis is true.

### Morpholino knockdown validation

*Fst*-expressing somite tissue from wild-type chick embryos was dissected and cultured in Dulbecco's modified Eagle medium, 10% foetal bovine serum and 1% penicillin/streptomycin for 4 h before transfecting with 1 mM translation-blocking FstMO (Gene Tools; sequence 5′-GATCCTCTGATTTAACATCCTCAGC-3′) or 1 mM StdMO negative control (Gene Tools; sequence 5′-CCTCTTACCTCAGTTACAATTTATA-3′) using Endoporter PEG (Gene Tools). Protein was extracted after 48 h. Protein lysate (30 μg) was run on pre-cast 4-15% polyacrylamide gels (Bio-Rad) and blotted onto polyvinylidene fluoride membrane (Bio-Rad). Primary antibody against Fst (Abcam ab47941; 1:2000) was applied at 4°C overnight and secondary polyclonal goat anti-rabbit-HRP (Cell Signaling Technology, 7074; 1:2000) was applied for 1 h at room temperature. Primary antibody against HSC70 (Santa Cruz, sc-7298; 1:2500) was applied at 4°C overnight and secondary polyclonal goat anti-mouse-HRP (Agilent, P0447; 1:1000) was applied for 1 h at room temperature. The blots were treated with an ECL substrate kit and imaged.

### *In ovo* embryo electroporation

Over-expression constructs encoding dnPax6 and Fst were constructed in pCIG (see Table S3 for PCR primers). Plasmid DNA (2-5 µg/μl) or plasmid DNA and FITC-labelled morpholino oligonucleotides (2 µg/μl and 0.5 mM, respectively) were injected into the open neural tube of stage HH8 chick embryos *in ovo* (Fig. 2A). A pair of platinum electrodes connected to an Ovodyne electroporator and current amplifier (Intracel) were then used to electroporate the DNA or DNA+morpholino into either the left or right side of the anterior neural tube via four pulses of 22 V and 50 ms duration at 1 s intervals. Once electroporated, embryos were sealed with adhesive tape and incubated for 10-12 h at 38°C until embryos had reached stage HH10.

### Whole-mount *in situ* hybridisation and immunofluorescence on sections

Embryos were fixed in 4% PFA overnight at 4°C, then dehydrated by methanol series and stored at −20°C. Following re-hydration, embryos were processed for whole-mount *in situ* hybridisation using 1 µg/ml DIG-labelled antisense probes for *Pax6 N-term* (Goulding et al., 1993), *Pax6 C-term*, *Vsx2*, *Mitf*, *Fst* (see Table S2 for PCR primers), *Tgfb2* (EST clone ChEST262a17) (Boardman et al., 2002), *Wnt2b* (a gift from Susan Chapman, Clemson University, SC, USA) and *Bmp4* (a gift from Elisa Martí, Institut de Biologia Molecular de Barcelona, Spain). Probes were hybridized at 65°C for up to 72 h. After incubation with 1:5000 anti-DIG antibody (Roche) and washing, 4.5 μl nitroblue tetrazolium (50 mg/ml) and 3.5 μl 5-bromo-4-chloro-3-indolyl phosphate (50 mg/ml) per 1.5 ml developing solution were used for colour development. Embryos were embedded in 7.5% gelatin, 15% sucrose and cryosectioned at 15 µm. Differences in morphology of sections are due to: (1) slight differences in staging of embryos between HH10− and HH10+; and (2) slight obliqueness and variation in the dorsal-ventral level of the horizontal sections. Following de-gelatinisation, sections were blocked in PBTS buffer (PBS with 2% BSA, 0.1% Triton X-100 and 10% goat serum) for 1 h at room temperature. EGFP transgene expression was then detected using rabbit anti-GFP primary antibody (Abcam, ab290; 1:500 dilution) and Alexa568 goat anti-rabbit secondary antibody (Invitrogen, A11036; 1:1000 dilution). Morpholino FITC fluorescence was observed directly. Labelled sections were imaged using a 20× objective on an Axioplan widefield fluorescence microscope with Axiocam HRc camera and Axiovision software (Carl Zeiss).

### Relative quantification of *in situ* hybridisation staining

Assuming that average cell size is invariant between left and right optic vesicles of the same embryo, then the relative area of staining is proportional to the relative number of cells exceeding a common detection threshold. To quantify this, bright-field micrographs were converted to greyscale, inverted then thresholded and the area of optic vesicle staining measured in FIJI (Schindelin et al., 2012). Transfected and contralateral controls from the same embryo were processed simultaneously to ensure identical treatment. Staining area in transfected vesicles was then normalised to internal contralateral controls, yielding a fold-change in gene expression area. Fold changes were log-transformed to bring data closer to a normal distribution (verified by Shapiro–Wilk test) prior to plotting and null hypothesis significance testing. Box plots showing mean Log10(fold change)±s.d. were generated in R with the package ‘Beeswarm’. A Welch's two-sample *t*-test (for pairwise comparisons) or one-way ANOVA with Tukey's post-hoc test (for groupwise comparisons) were used to calculate the probability of the observed (or more extreme) differences between sample means assuming that the null hypothesis is true.

### Reaction-diffusion simulations

Partial differential equations were coded in R using the function tran.1d() and tran.2d() from package ‘ReacTran’ to handle diffusion terms. 1D and 2D numerical simulations used the functions ode.1d() and ode.2d(), respectively, from package ‘deSolve’ and the default integrator. Parameter sweeps were performed to identify suitable diffusion rates (see Movies 1 and 2). 1D simulations were run with both periodic and zero-flux boundary conditions, with comparable results. 2D simulations were performed with zero-flux boundary conditions on explant-shaped domains, which best reflected experimental observations. See supplementary information for model code and narrative text. The model code is explained in the supplementary information, is available via our GitHub repository (https://github.com/GrocottLab/) and is accessible as an interactive Jupyter Notebook (https://mybinder.org/v2/gh/GrocottLab/Pax6-Fst-Tgfb2_Reaction_Diffusion_Models/master).

## Supplementary Material

Supplementary information

Reviewer comments
