# Supplementary material for: The Pax6 master control gene initiates spontaneous retinal development via a self-organising Turing network
Source: Development. 2020 Dec 23;147(24):dev185827. doi: 10.1242/dev.185827 (PMC7774904; doi:10.1242/dev.185827)
Supplement: Supplementary information [file develop-147-185827-s1.pdf]

Supplementary Information

1D simulation of Reaction-Diffusion Model A:

Introduction:

In this model, Pax6 drives expression of both Fst and Tgfb2. Fst monomers and Tgfb2 dimers may associate to form a hetero-tetrameric Fst:Tgfb2 complex. The Fst:Tgfb2 complex may dissociate in turn, thereby releasing Fst monomers and Tgfb2 dimers. Tgfb2 signals via association with Type I and Type II receptors, yielding an activated hetero-hexameric Tgfb2:Tgfbr signalling complex (comrpising one Tgfb2 ligand dimer and four Tgfb receptors - two Type I and two Type II; not shown here).

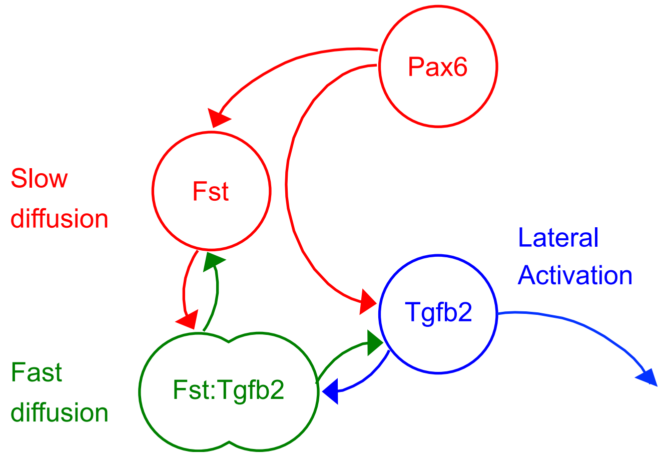

Figure S1. Summary of Model A in which Pax6 drives expression of both Fst and Tgfb2, whereas Fst inhibits Tgfb2 function via sequestration. Slow diffusion of Fst was postulated to result in local inhibition of Tgfb2 at the source of Pax6/Tgfb2/Fst expression. Conversely, fast diffusion of Tgfb2 was postulated to drive lateral activation of its downstream signalling pathway away from the Pax6/Fst/Tgfb2-expressing region.

As dicussed in the main article, Fst monomers are assumed to exhibit slower effective diffusion than Fst:Tgfb2 complexes, whereas effective diffusion of Tgfb2 dimers relative to Fst:Tgfb2 complexes is less critical. If this differential diffusion condition is met, Model A is able to elaborate an existing Pax6 pre-pattern (e.g. expression induced within the distal optic vesicle by upstream BMP signalling) via 'lateral activation' of Tgfb receptor complexes away from the source of Pax6 expression (e.g. within the adjacent proximal optic vesicle). In other words, the pattern of Tgfb pathway activation becomes the inverse of upstream Pax6 expression.

Development • Supplementary information

Equations:

The following system of equations formally represents the above interactions. Each equation describes the rate of change of concentration for the various molecular species downstream of Pax6. The initial concentrations of Pax6 (P) and Tgfb receptors (R) are held constant throughout the simulation, i.e. their rates of change, $\frac{\partial[P]}{\partial t}$ and $\frac{\partial[R]}{\partial t}$ are set to zero throughout.

Fst:

$$\frac{\partial[F]}{\partial t} = a_F.\frac{[P]}{[P]+1} - 2.a_{FT}.[F]^2.[T] + 2.b_{FT}.[FT] - b_F.[F] + D_F\frac{d^2[F]}{dx^2}$$

Where,

- Pax6 (P) positively regulates Fst expression via Michaelis-Menton kinetics $\frac{[P]}{[P]+1}$.
- Pairs of Fst monomers are consumed in the formation of Fst:Tgfb2 complexes (FT) via $-2.a_{FT}.[F]^2.[T]$, and released again via $+2.b_{FT}.[FT]$. This implements the two-way reaction: $1\,\mathrm{T} + 2\,\mathrm{F} \underset{b_{FT}}{\overset{a_{FT}}{\rightleftharpoons}} 1\,\mathrm{FT}$, whereby one Tgfb2 dimer reversibly associates with two Fst monomers.
- Fst is turned over via $-b_F.[F]$.
- Fst diffuses via $D_F\frac{d^2[F]}{dx^2}$.

Tgfb2:

$$\frac{\partial[T]}{\partial t} = \left(a_T.\frac{[P]}{[P]+1}\right)^2 - a_{FT}.[F]^2.[T] + b_{FT}.[FT] - a_{RT}.[R]^4.[T] - b_T.[T] + D_T\frac{d^2[T]}{dx^2}$$

Where,

- Pax6 (P) positively regulates Tgfb2 expression via Michaelis-Menton kinetics $\frac{[P]}{[P]+1}$.
- Tgfb2 is produced/secreted as a disulphide linked dimer, hence $\left(a_T.\frac{[P]}{[P]+1}\right)^2$.
- Tgfb2 is sequestered in the formation of Fst:Tgfb2 complexes (FT) via $-a_{FT}.[F]^2.[T]$, and released again via $+b_{FT}.[FT]$. This implements the two-way reaction: $1\,\mathrm{T} + 2\,\mathrm{F} \underset{b_{FT}}{\overset{a_{FT}}{\rightleftharpoons}} 1\,\mathrm{FT}$, whereby one Tgfb2 dimer reversibly associates with two Fst monomers.
- Tgfb2 is irreversibly consumed in the formation of activated receptor complexes (RT) via $-a_{RT}.[R]^4.[T]$. This implements the one-way reaction: $1\,\mathrm{T} + 4\,\mathrm{R} \xrightarrow{a_{RT}} 1\,\mathrm{RT}$, whereby one Tgfb2 dimer associates with four Tgfbr monomers (two type I receptors + two type II receptors). This reaction is irreverible since cells internalise and ultimately turn over activated receptor complexes.
- Tgfb2 is turned over via $-b_T.[T]$.
- Tgfb2 diffuses via $D_T\frac{d^2[T]}{dx^2}$.

Fst:Tgfb2 complex:

$$\frac{\partial[FT]}{\partial t} = a_{FT}.[F]^2.[T] - b_{FT}.[FT] + D_{FT}\frac{d^2[FT]}{dx^2}$$

Where,

- A pair of Fst monomers and a single Tgfb2 dimer associate via $a_{FT}.[F]^2.[T]$ forming a Fst:Tgfb2 complex (FT), which dissociates via $-b_{FT}.[FT]$. These terms implement the two-way reaction: $1\,\mathrm{T} + 2\,\mathrm{F} \underset{b_{FT}}{\overset{a_{FT}}{\rightleftharpoons}} 1\,\mathrm{FT}$.
- Fst:Tgfb2 diffuses via $D_{FT}\frac{d^2[FT]}{dx^2}$.

Tgfb2:Tgfbr signalling complex:

$$\frac{\partial [RT]}{\partial t} = a_{RT} . [R]^4 . [T] - b_{RT} . [RT]$$

Where,

- Active Tgfb2:Tgfbr signalling complexes (RT) are formed via $a_{RT} . [R]^4 . [T]$. This implements the one-way reaction: $1\,\mathrm{T} + 4\,\mathrm{R} \xrightarrow{a_{RT}} 1\,\mathrm{RT}$, whereby one Tgfb2 dimer associates with four Tgfbr monomers (two type I receptors + two type II receptors). This reaction is irreverible since cells internalise and ultimately turn over activated receptor complexes.
- Active Tgfb2:Tgfbr signalling complexes are turned over via $-b_{RT} . [RT]$.

R implementation:

The function 'modelA' implements the above system of equations and calculates the rates of change in concentration for each molecular species. The code breaks down into the following steps:

- Fetch the current system state (i.e. molecular concentrations for each 'cell' in the tissue).
- Evaluate the reaction terms for each molecular species.
- Evaluate diffusion terms for diffusible species. Diffusion terms are written for zero-flux boundary conditions. Terms for periodic boundaries are provided but commented out due to considerably longer execution times.
- Combine reaction and diffusion terms to determine rates of change for each species.
- Update a progress bar.
- Return the list of rates.

In [1]:
```
library(deSolve)
library(ReacTran)
library(viridis)
library(astro)
library(ggplot2)
library(readbitmap)

## ================
## Model equations
## ================
modelA <- function(time, state, parms) {
  with (as.list(parms), {

    # Generalise this: state[ ( (i-1)*N.A + 1) : (i * N.A) ]
    PAX6  <- state[1:N.A]
    TGFB2 <- state[(N.A+1):(2*N.A)]
    FST   <- state[(2*N.A+1):(3*N.A)]
    FT    <- state[(3*N.A+1):(4*N.A)]
    TGFBR <- state[(4*N.A+1):(5*N.A)]
    RT    <- state[(5*N.A+1):(6*N.A)]

    ## Reaction terms:
    reacPAX6   <- rep(0, N.A) # Concentration does not vary throughout simulati
on
    reacTGFB2 <- (
                  + (aTGFB2 * PAX6 / (PAX6 + 1) ) * (aTGFB2 * PAX6 / (PAX6 + 1
) )
                  - (2 * aFT * FST * FST * TGFB2 )
                  + (2 * bFT * FT)
                  - (aRT * TGFBR * TGFBR * TGFBR * TGFBR * TGFB2)
                  - (bTGFB2 * TGFB2)
                 )
    reacFST   <- (
                  + (aFST * PAX6) / ( (PAX6 + 1) )
                  - (2 * aFT * FST * FST * TGFB2)
                  + (2 * bFT * FT)
                  - (bFST * FST)
                 )
    reacFT    <- (
                  + (aFT * FST * FST * TGFB2)
                  - (bFT * FT)
                 )
    reacTGFBR <- rep(0, N.A) # Concentration does not vary throughout simulati
on
    reacRT    <- (
                  + (aRT * TGFBR * TGFBR * TGFBR * TGFBR * TGFB2)
                  - (bRT * RT)
                 )

    ## Diffusion terms - periodic boundary
    ## NOTE: these take longer to compute
    #diffTGFB2 <- tran.1D(C = TGFB2,
    #                     C.up = TGFB2[N.A],
    #                     C.down = TGFB2[1],
    #                     D = dTGFB2,
    #                     dx = xgridA)$dC
    #diffFST   <- tran.1D(C = FST,
    #                     C.up = FST[N.A],
    #                     C.down = FST[1],
    #                     D = dFST,
    #                     dx = xgridA)$dC
    #diffFT    <- tran.1D(C = FT,
    #                     C.up = FT[N.A],
    #                     C.down = FT[1],
    #                     D = dFT,
```

Development • Supplementary information

```
    #                        dx = xgridA)$dC

    ## Diffusion terms - zero-flux boundary
    diffTGFB2 <- tran.1D(C = TGFB2,
                         C.up = TGFB2[1],
                         C.down = TGFB2[N.A],
                         D = dTGFB2,
                         dx = xgridA)$dC
    diffFST   <- tran.1D(C = FST,
                         C.up = FST[1],
                         C.down = FST[N.A],
                         D = dFST,
                         dx = xgridA)$dC
    diffFT    <- tran.1D(C = FT,
                         C.up = FT[1],
                         C.down = FT[N.A],
                         D = dFT,
                         dx = xgridA)$dC

    ## Diffusion terms - fixed boundary
    #diffTGFB2 <- tran.1D(C = TGFB2,
    #                      C.up = 0,
    #                      C.down = 0,
    #                      D = dTGFB2,
    #                      dx = xgridA)$dC
    #diffFST   <- tran.1D(C = FST,
    #                      C.up = 0,
    #                      C.down = 0,
    #                      D = dFST,
    #                      dx = xgridA)$dC
    #diffFT    <- tran.1D(C = FT,
    #                      C.up = 0,
    #                      C.down = 0,
    #                      D = dFT,
    #                      dx = xgridA)$dC

    ## Rates of change = reaction  + diffusion:
    deltaPAX6          = reacPAX6
    deltaTGFB2         = reacTGFB2 + diffTGFB2
    deltaFST           = reacFST   + diffFST
    deltaFT            = reacFT    + diffFT
    deltaTGFBR         = reacTGFBR
    deltaRT            = reacRT

    setTxtProgressBar(pb, time)

    return (list(c(deltaPAX6, deltaTGFB2, deltaFST, deltaFT, deltaTGFBR, delta
RT)))
  })
}
```

```
Loading required package: rootSolve

Loading required package: shape

Loading required package: viridisLite

Loading required package: MASS

Loading required package: plotrix

Warning message:
“package ‘plotrix’ was built under R version 3.5.2”
```

Development • Supplementary information

Parameters:

In [2]:
```
## ==================
## Model parameters:
## ==================

## Space:
L.A      <- 50                             # Length of the 1D tissue
N.A      <- 100                            # Number of 1D 'cells'
dxA     <- L.A/N.A                         # Length of one 1D 'cell'
xgridA <- setup.grid.1D(N = N.A, x.down = L.A) # Output wanted at these cell p
ositions

## Time:
TimeA    <- 1500                  # Duration of the simulation
StepsA   <- 40                    # Number of time-steps
dtA      <-  TimeA/StepsA         # Size of each time-step
timesA  <- seq(0, TimeA, by = dtA) # Output wanted at these time intervals

## Molecuar Species
s.names = c("PAX6",
            "TGFB2 Dimer",
            "FST Monomer",
            "FST:TGFB2\nHeterotetramer",
            "TGFBR Monomer",
            "TGFB2:TGFBR\nComplex"
           )
SPECIES = length(s.names)

# Setup text progress bar - called by model function
pbA <- txtProgressBar(min = 0, max = TimeA, style = 3)

## Rate constants:
parmsA <- c(pb     <- pbA,     # Progress bar
            xgrid  <- xgridA,

            aPAX6  = 0.3,      # Production rate constant for PAX6
            bPAX6  = 0.1,      # Decay rate constant for PAX6

            aTGFB2 = 0.25,     # Production rate constant for TGFB2 dimer
            bTGFB2 = 0.1,      # Deacy rate constant for TGFB2 dimer
            dTGFB2 = 1,        # Diffusion rate constant for TGFB2 dimer

            aFST   = 1.5,      # Production rate constant for FST
            bFST   = 0.1,      # Decay rate constant for FST
            dFST   = 1,        # Diffusion rate constant for FST

            aFT    = 5,        # Association rate constant for FST:TGFB2 comple
x
            bFT    = 1,        # Dissociation rate constant for FST:TGFB2 compl
ex
            dFT    = 50,       # Diffusion rate constant for FST:TGBF2 complex

            aRT    = 20,       # Association rate constant for TGFBR:TGFB2 comp
lex
            bRT    = 0.1       # Decay rate constant for TGFBR:TGFB2 complex
           )
```

```
  |                                                                      |
0%
```

Development • Supplementary information

Initial Conditions:

The initial conditions describe a situation in which Pax6 expression is restricted to one region within the simulated tissue, corresponding to the distal optic vesicle. This is analagous to the situation in the embryo, where BMPs from the presumptive lens ectoderm promote Pax6 expression in the distal optic vesicle (see main text). Initial distributions of all other molecular species are uniform.

In [3]:
```
## ==================
## Initial conditions:
## ==================
stateA <- c(rep(0, N.A*(1.5/5)), rep(3, N.A*(2/5)), rep(0, N.A*(1.5/5)),  # PA
X6 pre-pattern
            rep(0, N.A),                                                   # TG
FB2
            rep(0, N.A),                                                   # FS
T
            rep(0, N.A),                                                   # FT
            rep(1, N.A),                                                   # TG
FBR
            rep(0, N.A) )                                                  # RT
```

Run Simulation:

In [4]:
```
## ==================
# Run the simulation:
## ==================
outA <- ode.1D(y        = stateA,
               times    = timesA,
               func     = modelA,
               parms    = parmsA,
               maxsteps = 1000000,
               nspec    = SPECIES,
               names    = s.names
              )

# Close the progress bar
close(pb)
```

```
  |======================================================================| 10
0%
```

Development • Supplementary information

In [5]:
```
## ==========================
## Plot the output via ggplot2
## ==========================
library(ggplot2)
print("Formatting simulation data...")
# Create data.frame for display data
x <- seq(from=dxA,to=N.A*dxA, by=dxA) # Length of tissue
t <- timesA                           # Time steps
s <- s.names                          # Species names
dataA <- expand.grid(X=x, Species=s, Time=t, Concentration= 0.0)

# Read simulation output into data.frame ready for display
# For each species...
for (s in seq(from=1,to=SPECIES,by=1)) {
    # And working backwards for each timepoint...
    for (t in timesA) {
        # Find column range in output for current species (timepoints are in s
eperate rows)
        s.start <- 2 + N.A * ( s - 1 )
        s.end   <- s.start + N.A - 1
        # Extract data for current timepoint (row) and species (columns)
        out.ACT <- outA[1+t/dtA, s.start:s.end]
        # Normalise for this species at this timepoint only
        #out.ACT <- out.ACT / max(out.ACT)
        # Find rows in data.frame for current species and timepoint
        s.index <- dataA$Species == s.names[s] # Rows for species
        t.index <- dataA$Time    == t        # Rows for timeopoint
        # Insert data for current species and timepoint
        dataA[s.index & t.index, 4] <- out.ACT
    }
    # Normalise across all timepoints for each species
    maximum <- max(dataA[s.index, 4])
    dataA[s.index, 4] <- dataA[s.index, 4] / maximum
}
print("...done!")

# Generate multi-faceted plot for endpoint...
print("Rendering endpoint plot...")
just.PAX6 <- dataA$Species == "PAX6"
just.RT   <- dataA$Species == s.names[6]
ggplot(dataA, aes(x=Time, y=X, z=Concentration)) +
geom_tile(aes(fill = Concentration ) ) +
facet_wrap(~ Species, ncol=3) +
theme_bw() +
labs(fill = "Normalised\nConcentration") +
ylab("Anterior-Posterior Axis (AU)") +
xlab("Time (AU)") +
theme(text = element_text(size=12)
     ) +
scale_fill_viridis(option="B")
print("...done!")
```

```
[1] "Formatting simulation data..."
[1] "...done!"
[1] "Rendering endpoint plot..."
[1] "...done!"
```

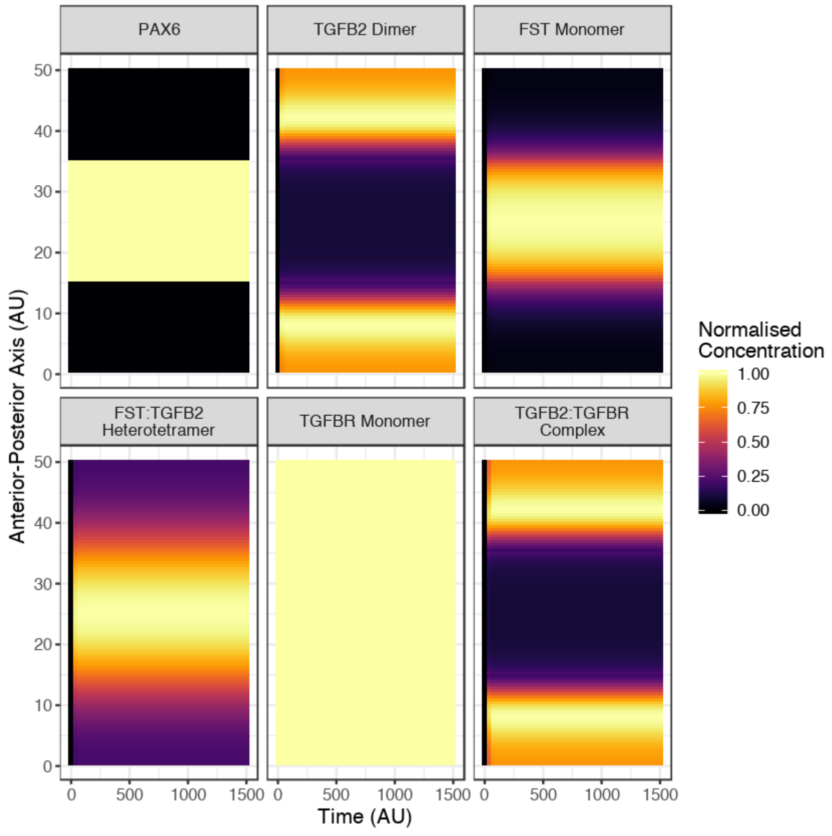

Figure S2. 1-D numerical simulation of Model A showing the normalised concentration distributions of each molecular species evolving over time. Brighter colours = higher concentrations.

Development • Supplementary information

1D simulation of Reaction-Diffusion Model B:

Introduction:

Model B is a direct extension of Model A, whereby Tgfb signalling inhibits Pax6's transcriptional activator function.

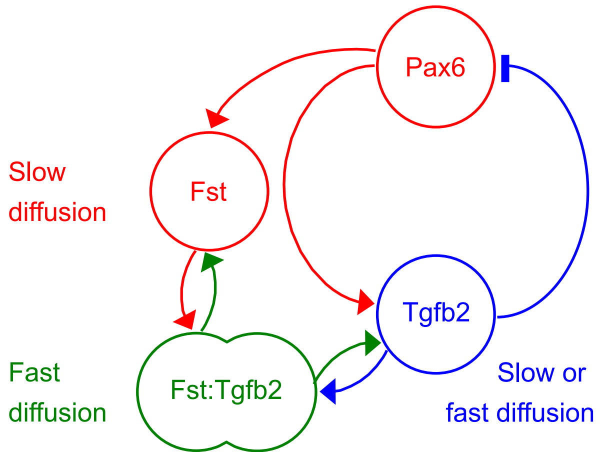

Figure S3. Summary of Model B in which Fst:Tgfb2 complex quickly diffuses and dissociates while Tgfb2 additionally inhibits Pax6 transcriptional activator function.

Note: In the following implementation of Model B Pax6 positively autoregulates its own expression. However, this is not strictly necessary for self-organisation to occur. This is because an indirect positive autoregulatory loop already exists; Pax6 acts via Fst to suppress Tgfb2's inhibition of Pax6 within the distal (*Pax6/Fst/Tgfb2*-expressing) pole. This indirect positive autoregulatory loop ("local autocatalysis") combined with lateral activation of Tgfb receptors ("long-range inhibition" of Pax6 function) is sufficient for self-organisation, though some parameter choices may require adjustment from those presented here.

Development • Supplementary information

Equations:

Pax6:

$$\frac{\partial[P]}{\partial t} = a_P.\frac{[P]}{[RT].[P]+1} - b_P.[P]$$

Where,

- Pax6 (P) positively autoregulates via Michaelis-Menton kinetics $\frac{[P]}{[P]+1}$.
- Activated Tgfbr complexes (RT) suppress Pax6 function via $\frac{[P]}{[RT].[P]+1}$.
- Pax6 is turned over via $-b_P.[P]$.

Tgfb2:

$$\frac{\partial[T]}{\partial t} = \left(a_T.\frac{[P]}{[RT].[P]+1}\right)^2 - a_{FT}.[F]^2.[T] + b_{FT}.[FT] - a_{RT}.[R]^4.[T] - b_T.[T] + D_T\frac{d^2[T]}{dx^2}$$

Where,

- Pax6 (P) positively regulates Tgfb2 expression via Michaelis-Menton kinetics $\frac{[P]}{[P]+1}$.
- Activated Tgfbr complexes (RT) suppress Pax6 function via $\frac{[P]}{[RT].[P]+1}$.
- Tgfb2 is produced as a dimer, hence $\left(a_T.\frac{[P]}{[RT].[P]+1}\right)^2$.
- Tgfb2 is sequestered in the formation of Fst:Tgfb2 complexes (FT) via $-a_{FT}.[F]^2.[T]$, and released again via $+b_{FT}.[FT]$. This implements the two-way reaction: $1\,\mathrm{T} + 2\,\mathrm{F} \underset{b_{FT}}{\overset{a_{FT}}{\rightleftharpoons}} 1\,\mathrm{FT}$, whereby one Tgfb2 dimer reversibly associates with two Fst monomers.
- Tgfb2 is irreversibly consumed in the formation of activated receptor complexes (RT) via $-a_{RT}.[R]^4.[T]$. This implements the one-way reaction: $1\,\mathrm{T} + 4\,\mathrm{R} \xrightarrow{a_{RT}} 1\,\mathrm{RT}$, whereby one Tgfb2 dimer associates with four Tgfbr monomers (two type I receptors + two type II receptors). This reaction is irreverible since cells internalise and ultimately turn over activated receptor complexes.
- Tgfb2 is turned over via $-b_T.[T]$.
- Tgfb2 diffuses via $D_T\frac{d^2[T]}{dx^2}$.

Fst:

$$\frac{\partial[F]}{\partial t} = a_F.\frac{[P]}{[RT].[P]+1} - 2.a_{FT}.[F]^2.[T] + 2.b_{FT}.[FT] - b_F.[F] + D_F\frac{d^2[F]}{dx^2}$$

Where,

- Pax6 (P) positively regulates Fst expression via Michaelis-Menton kinetics $\frac{[P]}{[P]+1}$.
- Activated Tgfbr complexes (RT) suppress Pax6 function via $\frac{[P]}{[RT].[P]+1}$.
- Pairs of Fst monomers are consumed in the formation of Fst:Tgfb2 complexes (FT) via $-2.a_{FT}.[F]^2.[T]$, and released again via $+2.b_{FT}.[FT]$. This implements the two-way reaction: $1\,\mathrm{T} + 2\,\mathrm{F} \underset{b_{FT}}{\overset{a_{FT}}{\rightleftharpoons}} 1\,\mathrm{FT}$, whereby one Tgfb2 dimer reversibly associates with two Fst monomers.
- Fst is turned over via $-b_F.[F]$.
- Fst diffuses via $D_F\frac{d^2[F]}{dx^2}$.

Fst:Tgfb2 complex:

$$\frac{\partial[FT]}{\partial t} = a_{FT}.[F]^2.[T] - b_{FT}.[FT] + D_{FT}\frac{d^2[FT]}{dx^2}$$

Development • Supplementary information

Where,

- A pair of Fst monomers and a single Tgfb2 dimer associate via $a_{FT}.[F]^2.[T]$ forming a Fst:Tgfb2 complex, which dissociates via $-b_{FT}.[FT]$. These terms implement the two-way reaction: $1\,\mathrm{T} + 2\,\mathrm{F} \underset{b_{FT}}{\overset{a_{FT}}{\rightleftharpoons}} 1\,\mathrm{FT}$.
- Fst:Tgfb2 diffuses via $D_{FT}\frac{d^2[FT]}{dx^2}$.

Activated Tgfbr complex:

$$\frac{\partial[RT]}{\partial t} = a_{RT}.[R]^4.[T] - b_{RT}.[RT]$$

R implementation:

The function 'modelB' implements the above system of equations and calculates the rates of change in concentration for each molecular species. The code breaks down into the following steps:

- Fetch the current system state (i.e. molecular concentrations for each 'cell' in the tissue).
- Evaluate the reaction terms for each molecular species.
- Evaluate diffusion terms for diffusible species. Diffusion terms are written for zero-flux boundary conditions. Terms for periodic boundaries are provided but commented out due to considerably longer execution times.
- Combine reaction and diffusion terms to determine rates of change for each species.
- Update a progress bar.
- Return the list of rates.

Development • Supplementary information

In [6]:
```
library(deSolve)
library(ReacTran)
library(viridis)
library(astro)
library(ggplot2)
library(readbitmap)

## ================
## Model equations
## ================
modelB <- function(time, state, parms) {
  with (as.list(parms), {

    # Generalise this: state[ ( (i-1)*N.B + 1) : (i * N.B) ]
    PAX6  <- state[1:N.B]
    TGFB2 <- state[(N.B+1):(2*N.B)]
    FST   <- state[(2*N.B+1):(3*N.B)]
    FT    <- state[(3*N.B+1):(4*N.B)]
    TGFBR <- state[(4*N.B+1):(5*N.B)]
    RT    <- state[(5*N.B+1):(6*N.B)]

    ## Reaction terms:
    reacPAX6   <- (
                  + (aPAX6 * PAX6) / (RT * PAX6 + 1)
                  - (bPAX6 * PAX6)
                 )
    reacTGFB2 <- (
                  + (aTGFB2 * PAX6 / ( RT * PAX6 + 1 ) ) ^ 2
                  - (aFT * FST * FST * TGFB2 )
                  + (bFT * FT)
                  - (aRT * TGFBR * TGFBR * TGFBR * TGFBR * TGFB2)
                  - (bTGFB2 * TGFB2)
                 )
    reacFST    <- (
                  + (aFST * PAX6) / (RT * PAX6 + 1)
                  - (2 * aFT * FST * FST * TGFB2)
                  + (2 * bFT * FT)
                  - (bFST * FST)
                 )
    reacFT     <- (
                  + (aFT * FST * FST * TGFB2)
                  - (bFT * FT)
                 )
    reacTGFBR <- rep(0, N.B) # Concentration does not vary throughout simulati
on
    reacRT     <- (
                  + (aRT * TGFBR * TGFBR * TGFBR * TGFBR * TGFB2)
                  - (bRT * RT)
                 )

    ## Diffusion terms - periodic boundary
    ## NOTE: these take longer to compute
    #diffTGFB2 <- tran.1D(C = TGFB2,
    #                     C.up = TGFB2[N.B],
    #                     C.down = TGFB2[1],
    #                     D = dTGFB2,
    #                     dx = xgridA)$dC
    #diffFST   <- tran.1D(C = FST,
    #                     C.up = FST[N.B],
    #                     C.down = FST[1],
    #                     D = dFST,
    #                     dx = xgridA)$dC
    #diffFT    <- tran.1D(C = FT,
    #                     C.up = FT[N.B],
    #                     C.down = FT[1],
```

```
    #                          D = dFT,
    #                          dx = xgridA)$dC

    ## Diffusion terms - zero-flux boundary
    diffTGFB2 <- tran.1D(C = TGFB2,
                         C.up = TGFB2[1],
                         C.down = TGFB2[N.B],
                         D = dTGFB2,
                         dx = xgridB)$dC
    diffFST   <- tran.1D(C = FST,
                         C.up = FST[1],
                         C.down = FST[N.B],
                         D = dFST,
                         dx = xgridB)$dC
    diffFT    <- tran.1D(C = FT,
                         C.up = FT[1],
                         C.down = FT[N.B],
                         D = dFT,
                         dx = xgridB)$dC

    ## Diffusion terms - fixed boundary
    #diffTGFB2 <- tran.1D(C = TGFB2,
    #                          C.up = 0,
    #                          C.down = 0,
    #                          D = dTGFB2,
    #                          dx = xgridA)$dC
    #diffFST   <- tran.1D(C = FST,
    #                          C.up = 0,
    #                          C.down = 0,
    #                          D = dFST,
    #                          dx = xgridA)$dC
    #diffFT    <- tran.1D(C = FT,
    #                          C.up = 0,
    #                          C.down = 0,
    #                          D = dFT,
    #                          dx = xgridA)$dC

    ## Rates of change = reaction  + diffusion:
    deltaPAX6          = reacPAX6
    deltaTGFB2         = reacTGFB2 + diffTGFB2
    deltaFST           = reacFST   + diffFST
    deltaFT            = reacFT    + diffFT
    deltaTGFBR         = reacTGFBR
    deltaRT            = reacRT

    setTxtProgressBar(pb, time)

    return (list(c(deltaPAX6, deltaTGFB2, deltaFST, deltaFT, deltaTGFBR, delta
RT)))
  })
}
```

Parameters:

Development • Supplementary information

In [7]:
```
## ==================
## Model parameters:
## ==================

## Space:
L.B       <- 50                                   # Length of the 1D tissue
N.B       <- 100                                  # Number of 1D 'cells'
dxB      <- L.B/N.B                               # Length of one 1D 'cell'
xgridB <- setup.grid.1D(N = N.B, x.down = L.B)   # Output wanted at these cell
 positions

## Time:
TimeB     <- 5000                      # Duration of the simulation
StepsB    <- 40                        # Number of time-steps
dtB       <- TimeB/StepsB              # Size of each time-step
timesB    <- seq(0, TimeB, by = dtB) # Output wanted at these time intervals

## Molecuar Species
s.names = c("PAX6",
            "TGFB2 Dimer",
            "FST Monomer",
            "FST:TGFB2\nHeterotetramer",
            "TGFBR Monomer",
            "TGFB2:TGFBR\nComplex"
           )
SPECIES = length(s.names)

# Setup text progress bar - called by model function
pbB <- txtProgressBar(min = 0, max = TimeB, style = 3)

## Rate constants:
parmsB <- c(pb     <- pbB,     # Progress bar
            xgrid  <- xgridB,

            aPAX6  = 0.3,      # Production rate constant for PAX6
            bPAX6  = 0.1,      # Decay rate constant for PAX6

            aTGFB2 = 0.25,     # Production rate constant for TGFB2 dimer
            bTGFB2 = 0.1,      # Deacy rate constant for TGFB2 dimer
            dTGFB2 = 1,        # Diffusion rate constant for TGFB2 dimer

            aFST   = 1.5,      # Production rate constant for FST
            bFST   = 0.1,      # Decay rate constant for FST
            dFST   = 1,        # Diffusion rate constant for FST

            aFT    = 5,        # Association rate constant for FST:TGFB2 comple
x
            bFT    = 1,        # Dissociation rate constant for FST:TGFB2 compl
ex
            dFT    = 50,       # Diffusion rate constant for FST:TGBF2 complex

            aRT    = 20,       # Association rate constant for TGFBR:TGFB2 comp
lex
            bRT    = 0.1       # Decay rate constant for TGFBR:TGFB2 complex
           )
```

```
  |                                                                      |
0%
```

Development • Supplementary information

Initial Conditions:

The initial conditions describe a situation in which Pax6 expression is restricted to one region within the simulated tissue, corresponding to the distal optic vesicle. This is analagous to the situation in the embryo, where BMPs from the presumptive lens ectoderm promote Pax6 expression in the distal optic vesicle (see main text). Initial distributions of all other molecular species are uniform.

In [8]:
```
## ==================
## Initial conditions:
## ==================
stateB <- c(jitter( rep(1, N.B), 0.01, 0.01), #PAX6 - no pre-pattern
            rep(0, N.B),                      # TGFB2
            rep(0, N.B),                      # FST
            rep(0, N.B),                      # FT
            rep(1, N.B),                      # TGFBR
            rep(0, N.B)                       # RT
           )
```

Run Simulation:

In [9]:
```
## ==================
# Run the simulation:
## ==================
outB <- ode.1D(y        = stateB,
               times    = timesB,
               func     = modelB,
               parms    = parmsB,
               maxsteps = 10000000,
               nspec    = SPECIES,
               names    = s.names
              )

# Close the progress bar
close(pb)
```

```
  |======================================================================| 10
0%
```

Development • Supplementary information

In [10]:
```
## ===========================
## Plot the output via ggplot2
## ===========================
library(ggplot2)
print("Formatting simulation data...")
# Create data.frame for display data
x <- seq(from=dxB,to=N.B*dxB, by=dxB) # Width of tissue
t <- timesB                           # Time steps
s <- s.names                          # Species names
dataB <- expand.grid(X=x, Species=s, Time=t, Concentration= 0.0)

# Read simulation output into data.frame ready for display
# For each species...
for (s in seq(from=1,to=SPECIES,by=1)) {
    # And working backwards for each timepoint...
    for (t in timesB) {
        # Find column range in output for current species (timepoints are in s
eperate rows)
        s.start <- 2 + N.B * ( s - 1 )
        s.end   <- s.start + N.B - 1
        # Extract data for current timepoint (row) and species (columns)
        out.ACT <- outB[1+t/dtB, s.start:s.end]
        # Normalise for this species at this timepoint only
        #out.ACT <- out.ACT / max(out.ACT)
        # Find rows in data.frame for current species and timepoint
        s.index <- dataB$Species == s.names[s] # Rows for species
        t.index <- dataB$Time    == t       # Rows for timeopoint
        # Insert data for current species and timepoint
        dataB[s.index & t.index, 4] <- out.ACT
    }
    # Normalise across all timepoints for each species
    maximum <- max(dataB[s.index, 4])
    dataB[s.index, 4] <- dataB[s.index, 4] / maximum
}
print("...done!")

# Generate multi-faceted plot for endpoint...
print("Rendering endpoint plot...")
just.PAX6 <- dataB$Species == "PAX6"
just.RT   <- dataB$Species == s.names[6]
ggplot(dataB, aes(x=Time, y=X, z=Concentration)) +
geom_tile(aes(fill = Concentration ) ) +
facet_wrap(~ Species, ncol=3) +
theme_bw() +
labs(fill = "Normalised\nConcentration") +
ylab("Anterior-Posterior Axis (AU)") +
xlab("Time (AU)") +
theme(text = element_text(size=12) ) +
scale_fill_viridis(option="B")
print("...done!")
```

Development • Supplementary information

```
[1] "Formatting simulation data..."
[1] "...done!"
[1] "Rendering endpoint plot..."
[1] "...done!"
```

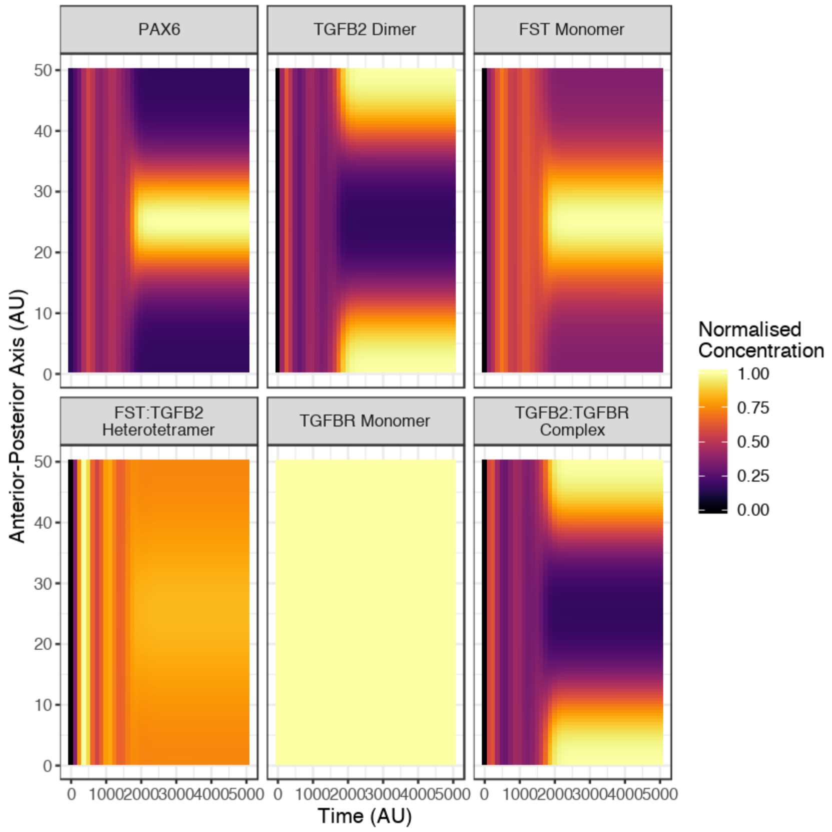

Figure S4. 1-D numerical simulation of Model B showing the normalised concentration distributions of each molecular species evolving over time. Brighter colours = higher concentrations.

Model C: 2D simulation of Reaction-Diffusion Model B:

This simulation is intended to mimic an optic vesicle explant cultured *in vitro*. The model implementation is re-wrtitten as function "modelC". The reaction terms are identical to Model B, but diffusion terms are re-written to facilitate diffusion in two dimensions within a domain of arbitrary shape. This arbitrary domain shape is loaded from a png image file within the Parameters setion. Diffusion occurs only within this domain (the diffusion multipliers DIFF.X and DIFF.Y equal 1 inside this domain and 0 outside), hence the boundary conditions are zero-flux.

Development • Supplementary information

In [11]:
```
library(deSolve)
library(ReacTran)
library(gganimate)
library(ggplot2)
library(gifski)
library(png)
library(viridis)
library(av)
library(raster)
library(readbitmap)

## ================
## Model equations
## ================
modelC <- function(time, state, parms) {
  with (as.list(parms), {
    # Generalise this: state[ ( (i-1)*N + 1) : (i * N) ]
    PAX6  <- DOMAIN * state[1:N]
    TGFB2 <- DOMAIN * state[(N+1):(2*N)]
    FST   <- DOMAIN * state[(2*N+1):(3*N)]
    FT    <- DOMAIN * state[(3*N+1):(4*N)]
    TGFBR <- DOMAIN * state[(4*N+1):(5*N)]
    RT    <- DOMAIN * state[(5*N+1):(6*N)]

    ## Reaction terms:
    reacPAX6  <- (
                 + (aPAX6 * PAX6) / (RT * PAX6 + 1)
                 - (bPAX6 * PAX6)
                )
    reacTGFB2 <- (
                 + (aTGFB2 * PAX6 / ( RT * PAX6 + 1 ) ) ^ 2
                 - (aFT * FST * FST * TGFB2 )
                 + (bFT * FT)
                 - (aRT * TGFBR * TGFBR * TGFBR * TGFBR * TGFB2)
                 - (bTGFB2 * TGFB2)
                )
    reacFST   <- (
                 + (aFST * PAX6) / (RT * PAX6 + 1)
                 - (2 * aFT * FST * FST * TGFB2)
                 + (2 * bFT * FT)
                 - (bFST * FST)
                )
    reacFT    <- (
                 + (aFT * FST * FST * TGFB2)
                 - (bFT * FT)
                )
    reacTGFBR <- rep(0, N.B) # Concentration does not vary throughout simulati
on
    reacRT    <- (
                 + (aRT * TGFBR * TGFBR * TGFBR * TGFBR * TGFB2)
                 - (bRT * RT)
                )

    ## Find boundaries:
    TGFB2.matrix <- matrix(data = TGFB2, nrow = X.N, ncol = Y.N)
    TGFB2.y.up   <- TGFB2.matrix[   , 1  ]
    TGFB2.y.down <- TGFB2.matrix[   , Y.N]
    TGFB2.x.up   <- TGFB2.matrix[1  ,    ]
    TGFB2.x.down <- TGFB2.matrix[X.N,    ]
    FST.matrix <- matrix(data = FST, nrow = X.N, ncol = Y.N)
    FST.y.up   <- FST.matrix[   , 1  ]
    FST.y.down <- FST.matrix[   , Y.N]
    FST.x.up   <- FST.matrix[1  ,    ]
    FST.x.down <- FST.matrix[X.N,    ]
    FT.matrix <- matrix(data = FT, nrow = X.N, ncol = Y.N)
```

Development • Supplementary information

```
    FT.y.up   <- FT.matrix[    , 1   ]
    FT.y.down <- FT.matrix[    , Y.N]
    FT.x.up   <- FT.matrix[1   ,     ]
    FT.x.down <- FT.matrix[X.N,      ]

    ### NO DIFFUSION AT DOMAIN BOUNDARIES = ZERO-FLUX BOUNDARY
    diffTGFB2 <- tran.2D(C = TGFB2.matrix, D.x = dTGFB2*DIFF.X, D.y = dTGFB2*D
IFF.Y, grid = grid_2d,
                            C.x.up = TGFB2.x.up, C.x.down = TGFB2.x.down,
                            C.y.up = TGFB2.y.up, C.y.down = TGFB2.y.down)$dC
    diffFST   <- tran.2D(C = FST.matrix,   D.x = dFST*DIFF.X,    D.y = dFST*DIF
F.Y,   grid = grid_2d,
                            C.x.up = FST.x.up,   C.x.down = FST.x.down,
                            C.y.up = FST.y.up,   C.y.down = FST.y.down   )$dC
    diffFT    <- tran.2D(C = FT.matrix,     D.x = dFT*DIFF.X,     D.y = dFT*DIF
F.Y,    grid = grid_2d,
                            C.x.up = FT.x.up,    C.x.down = FT.x.down,
                            C.y.up = FT.y.up,    C.y.down = FT.y.down    )$dC

    ### REMOVING DIFF.Y AND DIFF.X = DIFFUSION AT BOUNDARIES = FIXED BOUNDARY
    ### RESULTS IN CONCENTRIC STRIPES AS DOMAIN SIZE INCREASES
    ### THIS IS INCONSISTENT WITH OBSERVATIONS FROM EXPLANT EXPERIMENTS
    #diffTGFB2 <- tran.2D(C = TGFB2.matrix, D.x = dTGFB2, D.y = dTGFB2, grid =
grid_2d,
    #                        C.x.up = TGFB2.x.up, C.x.down = TGFB2.x.down,
    #                        C.y.up = TGFB2.y.up, C.y.down = TGFB2.y.down)$dC
    #diffFST   <- tran.2D(C = FST.matrix,    D.x = dFST,    D.y = dFST,    grid =
grid_2d,
    #                        C.x.up = FST.x.up,    C.x.down = FST.x.down,
    #                        C.y.up = FST.y.up,    C.y.down = FST.y.down   )$dC
    #diffFT    <- tran.2D(C = FT.matrix,     D.x = dFT,     D.y = dFT,     grid =
grid_2d,
    #                        C.x.up = FT.x.up,     C.x.down = FT.x.down,
    #                        C.y.up = FT.y.up,     C.y.down = FT.y.down    )$dC

    ## Rates of change = DOMAIN * (Reaction  + Diffusion):
    deltaPAX6          = DOMAIN * (reacPAX6)
    deltaTGFB2         = DOMAIN * (reacTGFB2 + diffTGFB2)
    deltaFST           = DOMAIN * (reacFST   + diffFST)
    deltaFT            = DOMAIN * (reacFT    + diffFT)
    deltaTGFBR         = DOMAIN * (reacTGFBR)
    deltaRT            = DOMAIN * (reacRT)

    setTxtProgressBar(pb, time)

    return (list(c(deltaPAX6, deltaTGFB2, deltaFST, deltaFT, deltaTGFBR, delta
RT)))
  })
}
```

```
Warning message:
“package ‘gganimate’ was built under R version 3.5.2”
Warning message:
“package ‘raster’ was built under R version 3.5.2”
Loading required package: sp


Attaching package: ‘raster’


The following object is masked from ‘package:gganimate’:

    animate


The following objects are masked from ‘package:MASS’:

    area, select
```

Parameters:

In [12]:

```
## ==================
## Model parameters:
## ==================

## Space:

# Read bitmap of 2D space
imgC <- read.bitmap("./OV_Explant4.png")
#imgC <- read.bitmap("./OV_Explant2.png")
# Extract dimension from bitmap
X.N <- dim(imgC)[1] #Number of elements (cells) across the width
Y.N <- dim(imgC)[2] #Number of elements (cells) across the height
N   <- X.N*Y.N      # Number of elements (cellS) in total
# Convert (#hex) bitmap to vector of integers
v <- as.integer(imgC)
# Convert this vector to matrix, just to check its dimensions
m <- matrix(v, X.N, Y.N)
# Convert matrix back to vector and use this as the model domain
DOMAIN <- as.vector(m)
# Use matrix to calculate which pixel/cell borders permit diffusion in X and Y
directions
DIFF.X <- rbind(rep(0.0, Y.N), floor( (m[-1,  ] + m[-nrow(m),        ]) / 2),
rep(0.0, Y.N) )
DIFF.Y <- cbind(rep(0.0, X.N), floor( (m[  ,-1] + m[        ,-ncol(m)]) / 2),
rep(0.0, X.N) )

X.L <- 13                                                  # Width  of the 2D tissue
Y.L <- 9                                                   # Height of the 2D tissue
dx  <- X.L/X.N                                             # Length of one 1D elemen
t in X
dy  <- Y.L/Y.N                                             # Length of one 1D elemen
t in Y
xgrid   <- setup.grid.1D(N = X.N, x.up = 0, L = X.L) # Output wanted at these
 X positions
ygrid   <- setup.grid.1D(N = Y.N, x.up = 0, L = Y.L) # Output wanted at these
 Y positions
grid_2d <- setup.grid.2D(xgrid, ygrid)               # Output wanted at these
 grid positions

## Time:
TimeC    <- 5000                               # Duration of the simulation
StepsC   <- 20                                 # Number of time-steps
dtC      <- TimeC/StepsC                       # Size of each time-step
timesC   <- seq(0, TimeC, by = dtC)            # Output wanted at these time in
tervals

## Molecuar Species
s.names = c("PAX6",
            "TGFB2 Dimer",
            "FST Monomer",
            "FST:TGFB2\nHeterotetramer",
            "TGFBR Monomer",
            "TGFB2:TGFBR\nComplex")
SPECIES = length(s.names)     # Count the number of species

# Setup text progress bar - called by model function
pbC <- txtProgressBar(min = 0, max = TimeC, style = 3)

## Rate constants:
parmsC <- c(pb     <- pbC,     # Progress bar

            aPAX6  = 0.3,      # Production rate constant for PAX6
            bPAX6  = 0.1,      # Decay rate constant for PAX6

            aTGFB2 = 0.25,     # Production rate constant for TGFB2 dimer
```

```
                bTGFB2 = 0.1,      # Deacy rate constant for TGFB2 dimer
                dTGFB2 = 1,        # Diffusion rate constant for TGFB2 dimer

                aFST   = 1.5,      # Production rate constant for FST
                bFST   = 0.1,      # Decay rate constant for FST
                dFST   = 1,        # Diffusion rate constant for FST

                aFT    = 5,        # Association rate constant for FST:TGFB2 comple
x
                bFT    = 1,        # Dissociation rate constant for FST:TGFB2 compl
ex
                dFT    = 50,       # Diffusion rate constant for FST:TGBF2 complex

                aRT    = 20,       # Association rate constant for TGFBR:TGFB2 comp
lex
                bRT    = 0.1       # Decay rate constant for TGFBR:TGFB2 complex
               )
```

```
  |                                                                      |
0%
```

Initial Conditions:

The initial conditions describe a situation in which Pax6 expression is graded from distal-high to proximal-low.

In [13]:

```
## ===================
## Initial conditions:
## ===================
Pax6.Grad <- rep(seq(from = 3,     to = 5, length.out = Y.N), X.N)
Pax6.Grad <- as.vector( t( matrix(Pax6.Grad, ncol = X.N, nrow = Y.N) ) )

stateC <- c(DOMAIN * Pax6.Grad,  # PAX6  - graded
            DOMAIN * rep(0, N),  # TGFB2
            DOMAIN * rep(0, N),  # FST
            DOMAIN * rep(0, N),  # FT
            DOMAIN * rep(1, N),  # TGFBR
            DOMAIN * rep(0, N)   # RT
           )
```

Run Simulation:

Development • Supplementary information

In [14]:
```
## ==================
# Run the simulation:
## ==================
cat("Initializing simulation...")
outputC <- ode.2D(y      = stateC,
                  times  = timesC,
                  func   = modelC,
                  parms  = parmsC,
                  nspec  = SPECIES,
                  names  = s.names,
                  dimens = c(X.N, Y.N),
                  method = "lsode",
                  lrw    = 7e+08
                 )
cat(" done!")
flush.console()
```

```
  |======================================================================| 10
0% done!
```

In [15]:
```
## =================
## Format the output
## =================
# Create data.frame for display data
x <- seq(from=dx, to=X.L,  by=dx) # Width of tissue
y <- seq(from=dy, to=Y.L,  by=dy) # Height of tissue
t <- timesC                        # Time steps
s <- s.names                       # Species names
dataC <- expand.grid(X=x, Y=y, Species=s, Time=t, Concentration= 0.0)
cat("Formatting output...")
# Read simulation output into data.frame ready for display
# For each species...
for (s in seq(from=1,to=SPECIES,by=1)) {
    # And for each subsequent timepoint...
    for (t in timesC) {
        # Find column range in output for current species (timepoints are in s
eperate rows)
        s.start <- 2 + N * ( s - 1 )
        s.end   <- s.start + N - 1
        # Extract data for current timepoint (row) and species (columns)
        out.ACT <- outputC[1+ (t/dtC), s.start:s.end]
        # Normalise for this species at this timepoint only
        #out.ACT <- out.ACT / max(out.ACT)
        # Find rows in data.frame for current species and timepoint
        s.index <- dataC$Species == s.names[s] # Rows for species
        t.index <- dataC$Time    == t          # Rows for timeopoint
        # Insert data for current species and timepoint
        dataC[s.index & t.index, 5] <- out.ACT
    }
    # Normalise across all timepoints for each species
    maximum <- max(dataC[s.index, 5])
    dataC[s.index, 5] <- dataC[s.index, 5] / maximum
}
cat("done!")
flush.console()
```

```
Formatting output...done!
```

In [16]:
```
## ===========================
## Plot the output via ggplot2
## ===========================
# Generate multi-faceted endpoint plot...
cat("Rendering plot...")
just.PAX6 <- dataC$Species == "PAX6"
p <- ggplot(dataC, aes(X, Y, z=Concentration, frame=Time)) +
geom_tile(aes(fill = Concentration ) ) +
facet_wrap(~ Species, ncol=3) +
theme_bw() +
theme(text = element_text(size=12) ) +
coord_fixed() +
scale_fill_viridis(option="B") +
ylab("Proximal-Distal Axis (AU)") +
xlab("Anterior-Posterior Axis (AU)") +
labs(title = "Normalised concentrations at endpoint") +
labs(fill = "Normalised\nConcentration")

plot(p)

cat("done!")
flush.console()
```

```
Rendering plot...done!
```

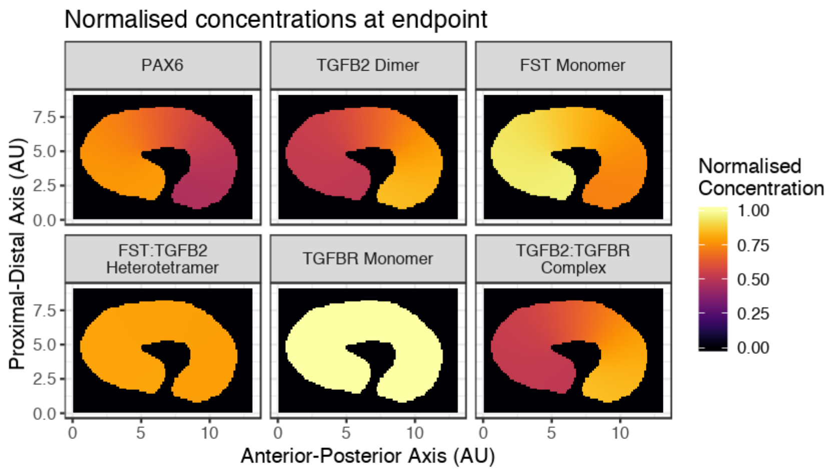

Figure S5. 2-D numerical simulation of Model C showing the normalised concentration distributions of each molecular species at endpoint (t = 5000). Brighter colours = higher concentrations.

In [17]:
```
## ===================================
## Plot output for Pax6 as aimated Gif
## ===================================
# Generate multi-faceted plot, animated over timepoints...
cat("Building plot...")
just.PAX6 <- dataC$Species == "PAX6"
p <- ggplot(dataC, aes(X, Y, z=Concentration, frame= Time)) +
geom_tile(aes(fill = Concentration ) ) +
facet_wrap(~ Species, ncol=3) +
theme_bw() +
theme(text = element_text(size=12) ) +
coord_fixed() +
scale_fill_viridis(option="B") +
transition_time(Time) + # Animate over timepoints
ylab("Proximal-Distal Axis (AU)") +
xlab("Anterior-Posterior Axis (AU)") +
labs(title = "Normalised concentrations at time = {frame_time}") +
labs(fill = "Normalised\nConcentration")

cat("done!")
flush.console()

cat("\nRendering plot...")

## Render animation as an ffmpeg video file and dispay
#anC1 <- animate(p,
#                fps=10,
#                nframes=2*StepsC,
#                width=720,
#                height=480,
#                start_pause=StepsC/2,
#                end_pause=StepsC/2,
#                rewind=FALSE,
#                renderer=av_renderer() )
#contents <- base64enc::base64encode(anC1)
#tag <- '<video src="data:video/mp4;base64,%s" controls autoplay></video>'
#IRdisplay::display_html(sprintf(tag, contents))

## Save ffmpeg video to file
#anim_save(animation = anC1, filename = "Model_C.mp4")

## Render animation as a GIF file and display
suppressMessages({
    anC2 <- gganimate::animate(p,
                               fps=10,
                               nframes=2*StepsC,
                               width=720,
                               height=480,
                               start_pause=StepsC/2,
                               end_pause=StepsC/2
                              )
})
contents <- base64enc::base64encode(anC2)
tag <- '<center><img src="data:image/gif;base64,%s"></center>'
IRdisplay::display_html(sprintf(tag, contents))

cat("done!")
flush.console()

## Save GIF animation to file
#anim_save(animation = anC2, filename = "Model_C.gif")
```

```
Building plot...done!
Rendering plot...
```

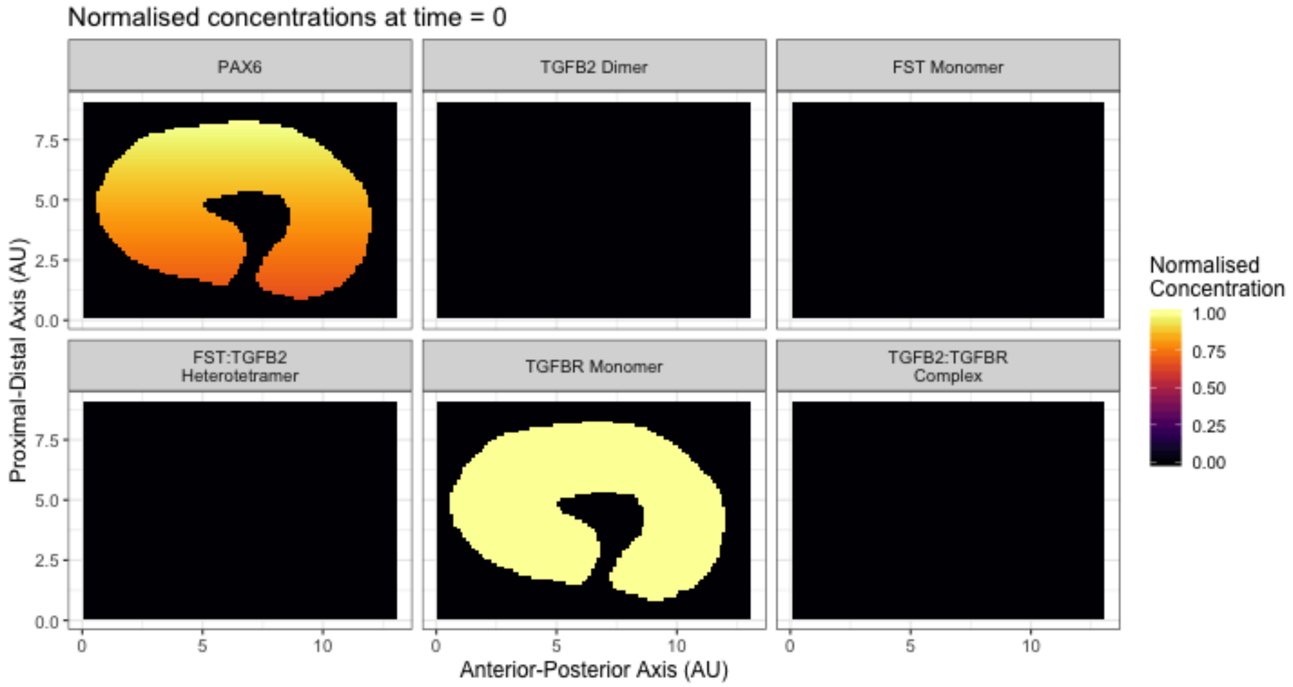


```
done!
```

Figure S6. 2-D numerical simulation of Model C showing the normalised concentration distributions of each molecular species evolving over time. In print/PDF form, this plot shows only the initial conditions for the simulation (i.e. at t = 0), while the endpoint (at t = 5000) is shown in Supp. Fig. S5. In the online Jupyter Notebook version (see methods section of paper for URL) this plot is animated. The animated plot is included as Supp. Movie 3. Brighter colours = higher concentrations.

Model D: Intrinsic SHH gradient restricts Pax6 re-polarisation to the former dorsal side

This model adds Shh as a 7th molecular species.

The governing equation for Pax6 is modified such that Pax6 expression is inhibited by Shh in addition to activated Tgfbr complexes.

Pax6:

$$\frac{\partial[P]}{\partial t} = a_P . \frac{[P]}{([RT] + [SHH]).[P] + 1} - b_P . [P]$$

Where,

- Pax6 (P) positively autoregulates via Michaelis-Menton kinetics $\frac{[P]}{[P]+1}$.
- Activated Tgfbr complexes (RT) and Shh (SHH) suppress Pax6 function via $\frac{[P]}{([RT]+[SHH]).[P]+1}$.
- Pax6 is turned over via $-b_P . [P]$.

The initial conditions are extended to include a Shh concentration gradient.

Development • Supplementary information

In [18]:
```
library(deSolve)
library(ReacTran)
library(gganimate)
library(ggplot2)
library(gifski)
library(png)
library(viridis)
library(av)
library(raster)
library(readbitmap)

## ================
## Model equations
## ================
modelD <- function(time, state, parms) {
  with (as.list(parms), {
    # Generalise this: state[ ( (i-1)*N + 1) : (i * N) ]
    PAX6   <- DOMAIN * state[(0*N+1):(1*N)]
    TGFB2  <- DOMAIN * state[(1*N+1):(2*N)]
    FST    <- DOMAIN * state[(2*N+1):(3*N)]
    FT     <- DOMAIN * state[(3*N+1):(4*N)]
    TGFBR  <- DOMAIN * state[(4*N+1):(5*N)]
    RT     <- DOMAIN * state[(5*N+1):(6*N)]
    SHH    <- DOMAIN * state[(6*N+1):(7*N)]

    ## Reaction terms:
    reacPAX6    <- (
                 + (aPAX6 * PAX6) / ( (RT + SHH) * PAX6 + 1)
                 - (bPAX6 * PAX6)
                )
    reacTGFB2 <- (
                 + (aTGFB2 * PAX6 / ( RT * PAX6 + 1 ) ) ^ 2
                 - (aFT * FST * FST * TGFB2 )
                 + (bFT * FT)
                 - (aRT * TGFBR * TGFBR * TGFBR * TGFBR * TGFB2)
                 - (bTGFB2 * TGFB2)
                )
    reacFST    <- (
                 + (aFST * PAX6) / (RT * PAX6 + 1)
                 - (2 * aFT * FST * FST * TGFB2)
                 + (2 * bFT * FT)
                 - (bFST * FST)
                )
    reacFT     <- (
                 + (aFT * FST * FST * TGFB2)
                 - (bFT * FT)
                )
    reacTGFBR <- rep(0, N.B) # Concentration does not vary throughout simulati
on

    ### For simulating Tgfb LOF, the production term may be multiplied by zero
###
    reacRT     <- (
                 + (aRT * TGFBR * TGFBR * TGFBR * TGFBR * TGFB2)
                 #+ (aRT * TGFBR * TGFBR * TGFBR * TGFBR * TGFB2) * 0
                 - (bRT * RT)
                )
    reacSHH    <- rep(0, N) # Concentration does not vary throughout simulation

    ## Find boundaries:
    TGFB2.matrix <- matrix(data = TGFB2, nrow = X.N, ncol = Y.N)
    TGFB2.y.up   <- TGFB2.matrix[    , 1   ]
    TGFB2.y.down <- TGFB2.matrix[    , Y.N]
    TGFB2.x.up   <- TGFB2.matrix[1   ,     ]
    TGFB2.x.down <- TGFB2.matrix[X.N,     ]
```

Development • Supplementary information

```
    FST.matrix <- matrix(data = FST, nrow = X.N, ncol = Y.N)
    FST.y.up   <- FST.matrix[    , 1   ]
    FST.y.down <- FST.matrix[    , Y.N]
    FST.x.up   <- FST.matrix[1   ,     ]
    FST.x.down <- FST.matrix[X.N,     ]
    FT.matrix <- matrix(data = FT, nrow = X.N, ncol = Y.N)
    FT.y.up   <- FT.matrix[    , 1   ]
    FT.y.down <- FT.matrix[    , Y.N]
    FT.x.up   <- FT.matrix[1   ,     ]
    FT.x.down <- FT.matrix[X.N,     ]

    ### NO DIFFUSION AT DOMAIN BOUNDARIES = ZERO-FLUX BOUNDARY
    diffTGFB2 <- tran.2D(C = TGFB2.matrix, D.x = dTGFB2*DIFF.X, D.y = dTGFB2*D
IFF.Y, grid = grid_2d,
                            C.x.up = TGFB2.x.up, C.x.down = TGFB2.x.down,
                            C.y.up = TGFB2.y.up, C.y.down = TGFB2.y.down)$dC
    diffFST   <- tran.2D(C = FST.matrix,   D.x = dFST*DIFF.X,   D.y = dFST*DIF
F.Y,   grid = grid_2d,
                            C.x.up = FST.x.up,   C.x.down = FST.x.down,
                            C.y.up = FST.y.up,   C.y.down = FST.y.down   )$dC
    diffFT    <- tran.2D(C = FT.matrix,    D.x = dFT*DIFF.X,    D.y = dFT*DIF
F.Y,    grid = grid_2d,
                            C.x.up = FT.x.up,    C.x.down = FT.x.down,
                            C.y.up = FT.y.up,    C.y.down = FT.y.down    )$dC

    ### REMOVING DIFF.Y AND DIFF.X = DIFFUSION AT BOUNDARIES = FIXED BOUNDARY
    ### RESULTS IN CONCENTRIC STRIPES AS DOMAIN SIZE INCREASES
    ### THIS IS INCONSISTENT WITH OBSERVATIONS FROM EXPLANT EXPERIMENTS
    #diffTGFB2 <- tran.2D(C = TGFB2.matrix, D.x = dTGFB2, D.y = dTGFB2, grid =
grid_2d,
    #                       C.x.up = TGFB2.x.up, C.x.down = TGFB2.x.down,
    #                       C.y.up = TGFB2.y.up, C.y.down = TGFB2.y.down)$dC
    #diffFST   <- tran.2D(C = FST.matrix,   D.x = dFST,   D.y = dFST,   grid =
grid_2d,
    #                       C.x.up = FST.x.up,   C.x.down = FST.x.down,
    #                       C.y.up = FST.y.up,   C.y.down = FST.y.down   )$dC
    #diffFT    <- tran.2D(C = FT.matrix,    D.x = dFT,    D.y = dFT,    grid =
grid_2d,
    #                       C.x.up = FT.x.up,    C.x.down = FT.x.down,
    #                       C.y.up = FT.y.up,    C.y.down = FT.y.down    )$dC

    ## Rates of change = DOMAIN * (Reaction  + Diffusion):
    deltaPAX6          = DOMAIN * (reacPAX6)
    deltaTGFB2         = DOMAIN * (reacTGFB2 + diffTGFB2)
    deltaFST           = DOMAIN * (reacFST   + diffFST)
    deltaFT            = DOMAIN * (reacFT    + diffFT)
    deltaTGFBR         = DOMAIN * (reacTGFBR)
    deltaRT            = DOMAIN * (reacRT)
    deltaSHH           = DOMAIN * (reacSHH)

    setTxtProgressBar(pb, time)

    return (list(c(deltaPAX6, deltaTGFB2, deltaFST, deltaFT, deltaTGFBR, delta
RT, deltaSHH)))
  })
}
```

Development • Supplementary information

In [19]:
```
## ==================
## Model parameters:
## ==================

### Space:

# Read bitmap of 2D space
#imgD <- read.bitmap("./OV_Explant4.png")
imgD <- read.bitmap("./OV_Explant2.png")
# Extract dimension from bitmap
X.N <- dim(imgD)[1] #Number of elements (cells) across the width
Y.N <- dim(imgD)[2] #Number of elements (cells) across the height
N   <- X.N*Y.N      # Number of elements (cellS) in total
# Convert (#hex) bitmap to vector of integers
v <- as.integer(imgD)
## Convert this vector to matrix, just to check its dimensions
m <- matrix(v, X.N, Y.N)
# Convert matrix back to vector and use this as the model domain
DOMAIN <- as.vector(m)
# Use matrix to calculate which pixel/cell borders permit diffusion in X and Y
directions
DIFF.X <- rbind(rep(0.0, Y.N), floor( (m[-1,  ] + m[-nrow(m),        ]) / 2),
rep(0.0, Y.N) )
DIFF.Y <- cbind(rep(0.0, X.N), floor( (m[  ,-1] + m[        ,-ncol(m)]) / 2),
rep(0.0, X.N) )

X.L <- 13                                                   # Width  of the 2D tissue
Y.L <- 9                                                    # Height of the 2D tissue
dx  <- X.L/X.N                                              # Length of one 1D elemen
t (cell) in X
dy  <- Y.L/Y.N                                              # Length of one 1D elemen
t (cell) in Y
xgrid   <- setup.grid.1D(N = X.N, x.up = 0, L = X.L) # Output wanted at these
 X positions
ygrid   <- setup.grid.1D(N = Y.N, x.up = 0, L = Y.L) # Output wanted at these
 Y positions
grid_2d <- setup.grid.2D(xgrid, ygrid)

## Time:
TimeD    <- 5000                                # Duration of the simulation
StepsD   <- 20                                  # Number of time-steps
dtD      <-  TimeC/StepsC                       # Size of each time-step
timesD   <- seq(0, TimeC, by = dtD)             # Output wanted at these time in
tervals

# Molecuar Species
s.names_D = c("PAX6",
              "TGFB2 Dimer",
              "FST Monomer",
              "FST:TGFB2\nHeterotetramer",
              "TGFBR Monomer",
              "TGFB2:TGFBR\nComplex",
              "SHH")
SPECIES_D = length(s.names_D)

# Setup text progress bar - called by model function
pbD <- txtProgressBar(min = 0, max = TimeC, style = 3)

# Rate constants:
parmsD <- c(pb     <- pbD,    # Progress bar

            aPAX6  = 0.3,     # Production rate constant for PAX6
            bPAX6  = 0.1,     # Decay rate constant for PAX6

            aTGFB2 = 0.25,    # Production rate constant for TGFB2 dimer
```

Development • Supplementary information

```
              bTGFB2 = 0.1,      # Deacy rate constant for TGFB2 dimer
              dTGFB2 = 1,        # Diffusion rate constant for TGFB2 dimer

              aFST   = 1.5,      # Production rate constant for FST
              bFST   = 0.1,      # Decay rate constant for FST
              dFST   = 1,        # Diffusion rate constant for FST

              aFT    = 5,        # Association rate constant for FST:TGFB2 comple
x
              bFT    = 1,        # Dissociation rate constant for FST:TGFB2 compl
ex
              dFT    = 50,       # Diffusion rate constant for FST:TGBF2 complex

              aRT    = 20,       # Association rate constant for TGFBR:TGFB2 comp
lex
              bRT    = 0.1       # Decay rate constant for TGFBR:TGFB2 complex
             )
```

```
  |                                                                      |
0%
```

Development • Supplementary information

In [26]:
```
## ==================
## Initial conditions:
## ==================

# Calculate the SHH gradient:
### For simulating Shh loss-of-function (LOF), SHH.X may be multiplied by zero
###
SHH.X <- 100 * dnorm(seq(1, X.N, by = 1), mean = X.N, sd = ( (2*X.N) / (X.L/2)
) )
#SHH.X <- 100 * dnorm(seq(1, X.N, by = 1), mean = 1,   sd = ( (2*X.N) / (X.L/
2) ) ) # Flip the gradient
#SHH.X <- SHH.X * 0
plot(SHH.X,
     xlab="Dorsal-Ventral Gradient",
     ylab="Shh Concentration",
     main="Shh Positional Information Gradient")

## ==================
## Initial conditions:
## ==================
Pax6.Grad <- rep(seq(from = 3,     to = 5, length.out = Y.N), X.N)
Pax6.Grad <- as.vector( t( matrix(Pax6.Grad, ncol = X.N, nrow = Y.N) ) )

stateD <- c(DOMAIN * Pax6.Grad,          # PAX6  - graded
            DOMAIN * rep(0, N),          # TGFB2
            DOMAIN * rep(0, N),          # FST
            DOMAIN * rep(0, N),          # FT
            DOMAIN * rep(1, N),          # TGFBR
            DOMAIN * rep(0, N),          # RT
            DOMAIN * rep(SHH.X, Y.N)     # SHH
           )
```

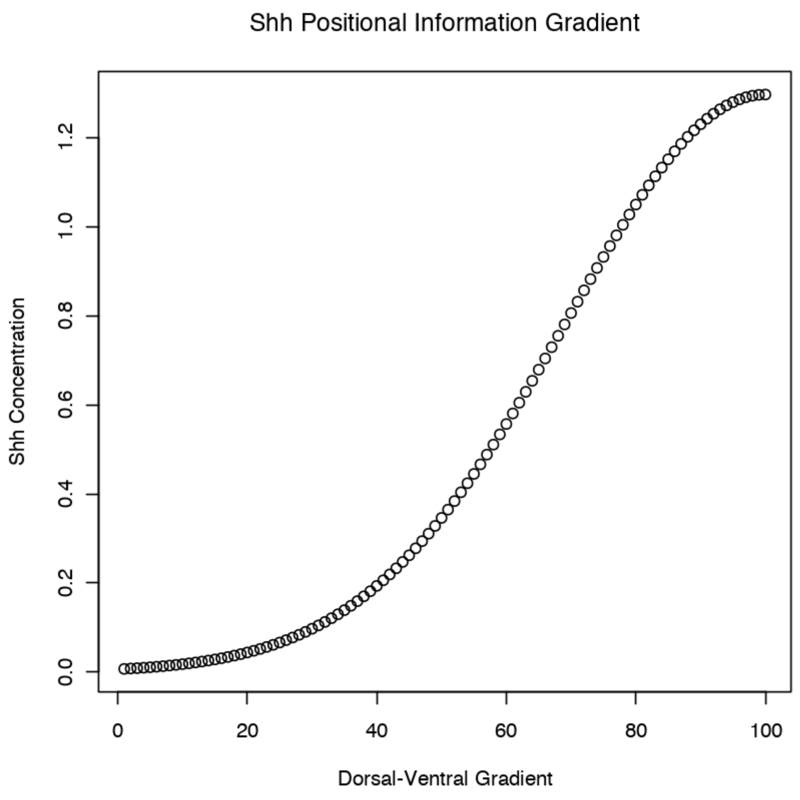

Figure S7. 1-D plot of the SHH positional information gradient used in Model D.

Development • Supplementary information

In [27]:

```
## ==================
# Run the simulation:
## ==================
cat("Initializing simulation...")
outputD <- ode.2D(y      = stateD,
                  times  = timesD,
                  func   = modelD,
                  parms  = parmsD,
                  nspec  = SPECIES_D,
                  names  = s.names_D,
                  dimens = c(X.N, Y.N),
                  method = "lsode",
                  lrw    = 7e+08)
cat(" done!")
flush.console()
```

```
|======================================================================   |  9
5% done!
```

In [28]:

```
## =================
## Format the output
## =================
# Create data.frame for display data
x <- seq(from=dx, to=X.L,  by=dx) # Width of tissue
y <- seq(from=dy, to=Y.L,  by=dy) # Height of tissue
t <- timesD                       # Number of time steps
s <- s.names_D                    # Number of species
dataD <- expand.grid(X=x, Y=y, Species=s, Time=t, Concentration= 0.0)
cat("Formatting output...")
# Read simulation output into data.frame ready for display
# For each species...
for (s in seq(from=1,to=SPECIES_D,by=1)) {
    # And for each subsequent timepoint...
    for (t in timesD) {
        # Find column range in output for current species (timepoints are in s
eperate rows)
        s.start <- 2 + N * ( s - 1 )
        s.end   <- s.start + N - 1
        # Extract data for current timepoint (row) and species (columns)
        out.ACT <- outputD[1+ (t/dtD), s.start:s.end]
        # Normalise for this species at this timepoint only
        #out.ACT <- out.ACT / max(out.ACT)
        # Find rows in data.frame for current species and timepoint
        s.index <- dataD$Species == s.names_D[s]  # Rows for species
        t.index <- dataD$Time    == t#*dt         # Rows for timeopoint
        # Insert data for current species and timepoint
        dataD[s.index & t.index, 5] <- out.ACT
    }
    # Normalise across all timepoints for each species
    maximum <- max(dataD[s.index, 5])
    dataD[s.index, 5] <- dataD[s.index, 5] / maximum
}
cat("done!")
flush.console()
```

```
Formatting output...done!
```

In [29]:
```
## =================
## Plot the output
## =================
# Generate multi-faceted endpoint plot...
cat("Rendering plot...")
ggplot(dataD, aes(X, Y, z=Concentration, frame=Time)) +
geom_tile(aes(fill = Concentration ) ) +
facet_wrap(~ Species, ncol=3) +
theme_bw() +
theme(text = element_text(size=8) ) +
coord_fixed() +
scale_fill_viridis(option="B") +
ylab("Proximal-Distal Axis (AU)") +
xlab("Dorsal-Ventral Axis (AU)") +
labs(title = "Normalised concentrations at endpoint") +
labs(fill = "Normalised\nConcentration")

cat("done!")
flush.console()
```

```
Rendering plot...done!
```

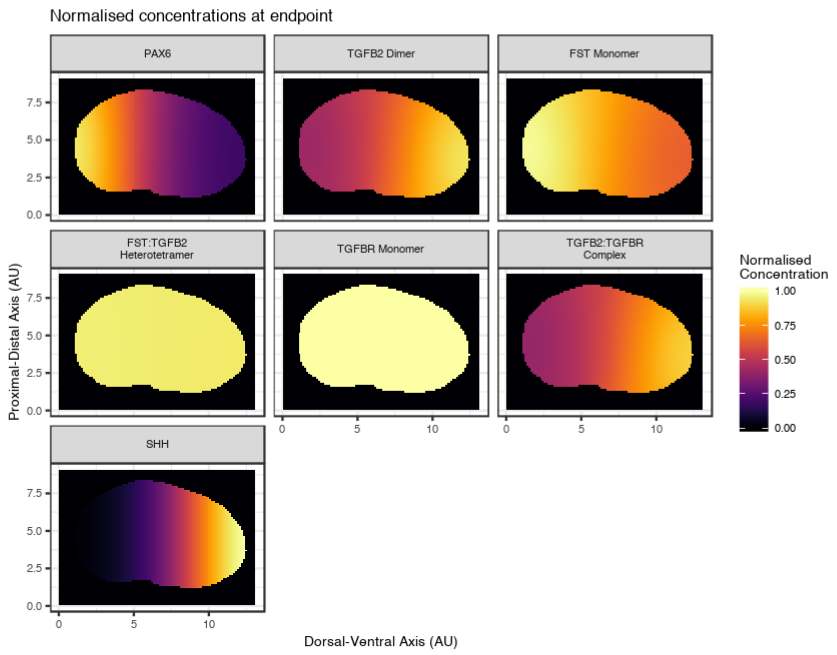

Figure S8. 2-D numerical simulation of Model D showing the normalised concentration distributions of each molecular species at endpoint (t = 5000). Brighter colours = higher concentrations.

Development • Supplementary information

In [30]:
```
## =================
## Plot the output
## =================
# Generate multi-faceted plot, animated over timepoints...
cat("Building plot...")
p <- ggplot(dataD, aes(X, Y, z=Concentration, frame= Time)) +
geom_tile(aes(fill = Concentration ) ) +
facet_wrap(~ Species, ncol=3) +
theme_bw() +
theme(text = element_text(size=12) ) +
coord_fixed() +
scale_fill_viridis(option="B") +
transition_time(Time) + # Animate over timepoints
ylab("Proximal-Distal Axis (AU)") +
xlab("Dorsal-Ventral Axis (AU)") +
labs(title = "Normalised concentrations at time = {frame_time}") +
labs(fill = "Normalised\nConcentration")

cat("done!")
flush.console()

cat("\nRendering plot...")

## Render animation as an ffmpeg video file and dispay
#anD1 <- animate(p,
#                 fps=10,
#                 nframes=2*StepsD,
#                 width=720,
#                 height=480,
#                 start_pause=StepsD/2,
#                 end_pause=StepsD/2,
#                 rewind=FALSE,
#                 renderer=av_renderer() )
#contents <- base64enc::base64encode(anD1)
#tag <- '<video src="data:video/mp4;base64,%s" controls autoplay></video>'
#IRdisplay::display_html(sprintf(tag, contents))

## Save ffmpeg video to file
#anim_save(animation = anD1, filename = "Model_D.mp4")

## Render animation as a GIF file and display
suppressMessages({
    anD2 <- gganimate::animate(p,
                              fps=10,
                              nframes=2*StepsD,
                              width=720,
                              height=480,
                              start_pause=StepsD/2,
                              end_pause=StepsD/2)
})
contents <- base64enc::base64encode(anD2)
tag <- '<center><img src="data:image/gif;base64,%s"></center>'
IRdisplay::display_html(sprintf(tag, contents))

cat("done!")
flush.console()

## Save GIF animation to file
#anim_save(animation = anD2, filename = "Model_D.gif")
```

```
Building plot...done!
Rendering plot...
```

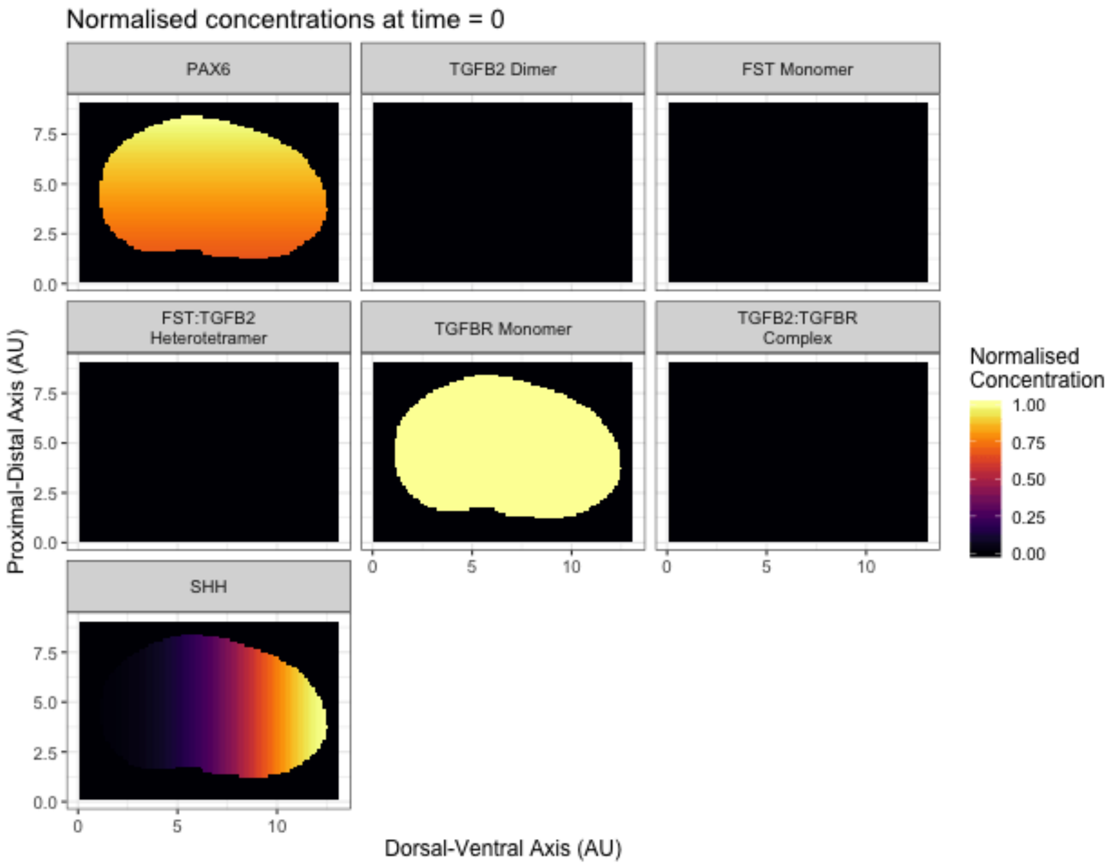


```
done!
```

Figure S9. 2-D numerical simulation of Model D showing the normalised concentration distributions of each molecular species evolving over time. In print/PDF form, this plot shows only the initial conditions for the simulation (i.e. at t = 0), while the endpoint (at t = 5000) is shown in Supp. Fig. S8. In the online Jupyter Notebook version (see methods section of paper for URL) this plot is animated. Brighter colours = higher concentrations.

```
In [ ]:
```

Development • Supplementary information

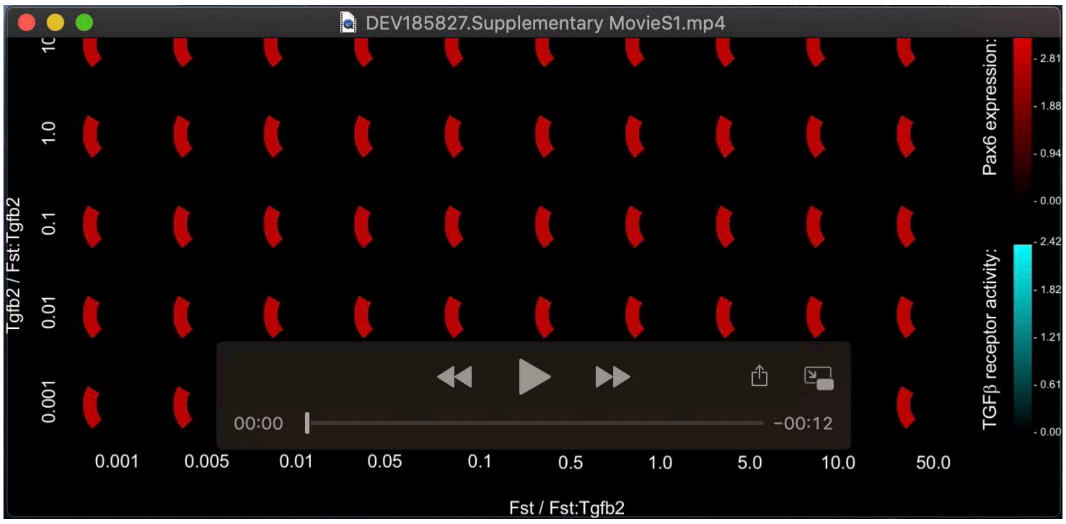

Movie 1. 1-D numerical simulations of Model A. Simulations demonstrate that local inhibition and lateral-activation of Tgfb2 signalling (i.e. Tgfb2:Tgfbr complex formation) may occur if Fst:Tgfb2 hetero-tetramer diffusion exceeds that of Fst monomers. Pax6 ‘expression’ was restricted to just the left side of a circular tissue throughout. Simulations were performed using different relative diffusion rates for Fst monomers (x-axis) and Tgfb2 dimers (y-axis), while Fst:Tgfb2 hetero-tetramer diffusion rate was held constant. Simulations are shown three times; first with both Pax6 (red) and activated Tgfb receptor (i.e. Tgfb2:Tgfbr complex; cyan) concentrations together; second with just activated receptor concentrations; third with just Pax6 concentrations.

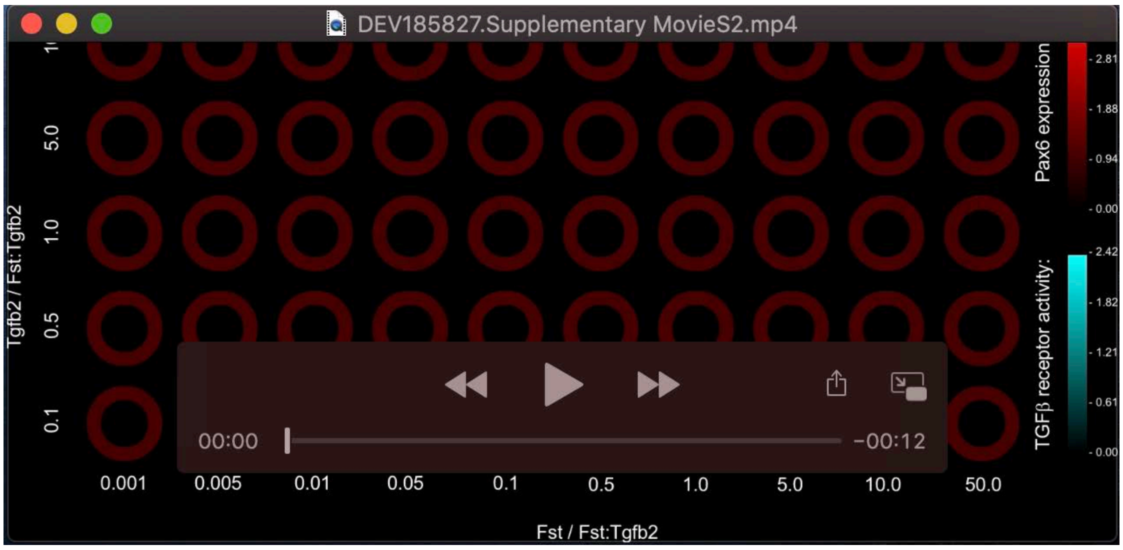

Movie 2. 1-D numerical simulations of Model B. Simulations demonstrate that the *Pax6*/ *Fst*/*Tgfb2* network of Model B could spontaneously polarise the optic vesicle into $Pax6^{+}$ and $Pax6^{-}$ 'poles' if Fst:Tgfb2 hetero-tetramer diffusion exceeds that of Fst monomers. Pax6 was initially distributed throughout a circular tissue, but with small local fluctuations. Simulations were performed using different relative diffusion rates for Fst monomers (x-axis) and Tgfb2 dimers (y-axis), while Fst:Tgfb2 hetero-tetramer diffusion rate was held constant. Simulations are shown three times; first with both Pax6 (red) and activated Tgfb receptor (i.e. Tgfb2:Tgfbr complex; cyan) concentrations together; second with just activated receptor concentrations; third with just Pax6 concentrations.

Development • Supplementary information

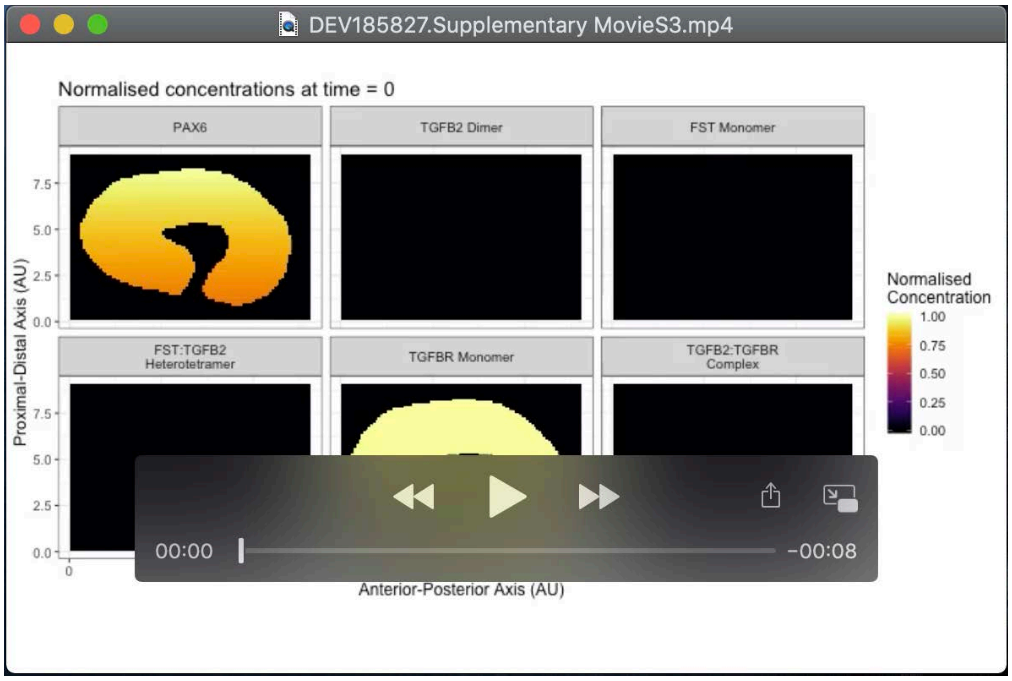

Movie 3. 2-D numerical simulation of Model C. This movie shows a 2-D numerical simulation of Model C within an explant-shaped domain. The initial distal-high to proximal-low Pax6 pattern is dynamically re-polarised, generating a graded expression along the explant's longest axis. This long axis could represent the optic vesicle's former Anterior-Posterior axis (as labelled) or Dorsal-Ventral axis. The movie shows the evolving concentration patterns of all six molecular species represented in the model.

Development • Supplementary information

Table S1. Primer sequences for qRT-PCR.

Target Gene	Upstream (5' – 3')	Downstream (5' – 3')
β-actin	CCAGCTGGGAGGAGCCGGT	CTGGGGAACACAGCCCGCTT
Pax6	AGTGGAGAAGGGAGGAGAAG	TGATGTGAAAGAGGAAACGGG
Vsx2	CACTACCCTGATGTGTATGCTAG	CCCATACTCAGCCATCACG
Wnt2b	TGAGTGCCAGTACCAATTCC	GAGATGGCGTAGACGAAGG
Mitf	AAGAACTGGGCACCTTGATAC	TGTCTGTTCTCAAGTTCCTTCG
Tgfb2	TTGATGTCACTGAGGCTGTAC	CAAATCTTGCTTCAGGCTCC
Fst	TGAAAGCAAGTCAGATGAGGC	CCTCTGGGTCTTCGTTAATGG

Table S2. Primer sequences for PCR cloning of in situ hybridization probes.

Target Gene	Upstream (5' – 3')	Downstream (5' – 3')
Vsx2	CTTCCCTCAACCAGAGCAAG	CATGGCATCTGCAAGCTAAA
Mitf	GGGATCCAAACTGGAAGACATC	CCTTTCAAGTGTTTACAAAGAACG
Fst	CAGGCTGGGAACTGCTGG	ACAGGCTGCCTCTTTCAT
Pax6 C-term	AGGAGGGGAGAACACCAACT	GGGAAATGAGACCTGTGGAA

Table S3. Primer sequences for PCR cloning of expression constructs.

Gene	Upstream (5' – 3')	Downstream (5' – 3')
dnPax6	GCCATCGATATGCAGAACAG	TTACTTTTCGCTAGCCAGGTTG
Fst-HA	ATGTTAAATCAGAGGATCCACCCG	CCACTCTAGAATGGAAGATATAGGAAAGC

Development • Supplementary information
