# Supplementary material for: The Pax6 master control gene initiates spontaneous retinal development via a self-organising Turing network
Source: Development. 2020 Dec 23;147(24):dev185827. doi: 10.1242/dev.185827 (PMC7774904; doi:10.1242/dev.185827)
Supplement: Reviewer comments [file develop-147-185827_review_history.pdf]

The *Pax6* master control gene initiates spontaneous retinal development via a self-organising Turing network

Timothy Grocott, Estefania Lozano-Velasco, Gi Fay Mok and Andrea E. Münsterberg
DOI: 10.1242/dev.185827

Editor: James Briscoe

Review timeline

Original submission:	23 October 2019
Editorial decision:	23 December 2019
First revision received:	29 October 2020
Editorial decision:	4 November 2020
Second revision received:	4 November 2020
Accepted:	5 November 2020

Original submission

First decision letter

MS ID#: DEVELOP/2019/185827

MS TITLE: The Pax6 master control gene initiates spontaneous retinal development via a self-organising Turing network

AUTHORS: Timothy Grocott, Estefania Lozano Velasco, Gi Fay Mok, and Andrea E Munsterberg

I apologise for the delay in reviewing your study. It was difficult to find reviewers. However, have now received all the referees' reports on the above manuscript, and have reached a decision. The referees' comments are appended below, or you can access them online: please go to BenchPress and click on the 'Manuscripts with Decisions' queue in the Author Area.

As you will see, the referees express considerable interest in your work, but have some significant criticisms and recommend a substantial revision of your manuscript before we can consider publication. Both reviewers highlight the novelty of your study but wish to see further evidence that the proposed network is responsible for pattern formation. Reviewer 1 suggests experiments that generate mosaic perturbations and then assessment of non-autonomous effects. These appears to be a good test of the model. Reviewer 2 suggests incorporating single cell RNA-seq, I think this is beyond the scope of your current study. Nevertheless, I'd ask that you to respond to the other comments made by this reviewer.

If you are able to revise the manuscript along the lines suggested, which may involve further experiments, I will be happy receive a revised version of the manuscript. Your revised paper will be re-reviewed by one or more of the original referees, and acceptance of your manuscript will depend on your addressing satisfactorily the reviewers' major concerns. Please also note that Development will normally permit only one round of major revision.

Please attend to all of the reviewers' comments and ensure that you clearly highlight all changes made in the revised manuscript. Please avoid using 'Tracked changes' in Word files as these are lost in PDF conversion. I should be grateful if you would also provide a point-by-point response detailing how you have dealt with the points raised by the reviewers in the 'Response to Reviewers' box. If you do not agree with any of their criticisms or suggestions please explain clearly why this is so.

© 2020. Published by The Company of Biologists under the terms of the Creative Commons Attribution License (https://creativecommons.org/licenses/by/4.0/).

Reviewer 1

Advance summary and potential significance to field

Grocott et al propose that a Turing-like network, comprising Pax6/Fst/Tgfb2, is involved in polarising the optic vesicle along its proximal-distal axis. They suggest that in vivo this network cooperates with external signals like BMP to polarise the tissue, but that this network also allows polarity to self-organize as had been observed in retinal organoids.
If convincingly shown, I would regard this hypothesis as highly significant and of broad interest to the developmental biology community.

Many aspects of this proposal are well supported by the data provided. In particular, the authors show that interactions between Pax6, Fst and Tgfb2 are consistent with the logic required for a Turing system (summarized in Fig. 4d), and very nicely use simulations to argue that this putative Turing network is sufficient to explain optic vesicle polarization in silico.

Whilst the evidence for each of the interactions in Fig. 4d is good, I think that further evidence is required to show that this network is responsible (necessary? sufficient?) for optic vesicle polarization in vivo or in vitro. In other words, I am convinced that this network exists, but given the data presented, remain unconvinced that it is the primary driver of self-organization.

Comments for the author

MAJOR COMMENTS
1. FURTHER EVIDENCE FOR THE ROLE OF PAX6/FST/TGFB2 NETWORK IN OPTIC VESICLE POLARIZATION

I think there are a number of perturbation experiments that could more directly support the hypothesis that the role of the Pax6/Fst/Tgfb2 network is to spontaneously polarize the optic vesicle. However, one would want to see a change in the patterning (e.g. via pax6 expression), that is more subtle than either that pax6 is downregulated (e.g. Fig 5e) or pax6 is unaffected (e.g. Fig 5i).

One possibility I could imagine would be to perform some mosaic perturbation (similar to Fig 5), and show that this can change pax6 patterning throughout the optic vesicle. It would be important to see non-cell-autonomous changes. For example, could the authors use mosaic FstMO or mosaic Fst overexpression (data they may already have) to ask whether they can shift the location of the pax6 domain? The results of these experiments could then be compared to simulations in which the perturbation is applied in silico.

The experimental design in Fig 5g,h could also provide complementary evidence, if I understand it correctly. Here, endogeneous Fst is blocked by the morpholino, but constitutive (i.e. pax6-independent) Fst is provided. This is a nice experiment because it doesn't completely remove Fst, but only removes its transcriptional regulation (including via pax6), which (my intuition suggests) will be important for the self-organizing abilities of the network. From the data presented (Fig 5g), it appears that the vesicle polarises fine – is this to be expected from the simulations?

2. BETTER DESCRIPTION OF TISSUE GEOMETRY

Throughout the manuscript, I was not always clear on the geometry/orientation of the data presented – both experimental and simulations. I would recommend:

a) early on, provide a brief description and schematic of the 3D structure of the optic vesicle, the proximal-distal patterning events and their relation to the vesicle-to-cup transition. Explain how this fits in with the orientation of the in situs presented. (Also: it appears that the morphology varies between different sections, why is this?)

b) When describing the simulations, can you include a longer discussion in the main text on tissue geometry and boundary conditions. In the supplementary movies, it looks like there are 1D domains

© 2020. Published by The Company of Biologists under the terms of the Creative Commons Attribution License (https://creativecommons.org/licenses/by/4.0/).

with periodic boundary conditions. Can the authors justify this choice (or at least discuss it in more detail, particularly from a biological standpoint)?

MINOR POINTS
1. Can you give more details on the Fst transgene (as used in Fig 5g,h)?
2. Lines 15-16, page 11: it is unclear a priori that log-transforming guarantees normality for fold changes – you have to make further assumptions (or show/state that log transforming makes your data closer to normally distributed).
3. Fig 4k-m are not like-for-like comparisons. In the simulations, patterns are presented; whereas in the experimental data, overall levels of expression are presented. It is hard to compare the two. Can the patterning of the tissues in Fig 4m be assayed? If not, can the simulations be used to generate an in silico version of Fig 4m?
4. In the supplementary movies (and in Fig 4e), brief oscillations in Pax6 are seen – is this seen in vivo/in vitro? Is this a by-product of the initial conditions chosen?
5. When discussing the data from Fig 4g-m, it should be emphasized that these predictions (i.e. larger domain gives multiple poles, smaller domain can result in no patterning) is a rather general feature of Turing systems and not a specific test of the Pax6/Fst/Tgfb2 network being responsible.

Reviewer 2

Advance summary and potential significance to field

The manuscript by Timothy Grocott and co-workers tackles a key question of developmental biology, the pattern formation in early vertebrate embryos. They use patterning of early optic chick vesicle as an experimental model. Their central hypothesis is that a lineage-specific DNA-binding transcription factor Pax6 is a cornerstone of a cross-talk of multiple signaling pathways and these are wired together as a putative Turing network. The experiments test this model through three components of TGF/BMP signaling, Bmp4 follistatin (Fst), and Tfgb2 at their mRNA levels. The main findings in the paper are summarized in a model that accounts for differences of Pax6 regulation in distal and proximal regions of the optic vesicle (Fig. 6).
Overall, these findings are novel and within the scope of journal Development.

Comments for the author

There is no direct relationship established between the Pax6 and Vsx2 expression in the retina. Additional genes, such as Fgf9 and Erk1/2, should be examined. Auto-regulation of Pax6 in the optic vesicle was probed through dnPax6 mis-expression. This experiment requires additional data to determine levels of ectopic expression in relationship to their endogenous levels. Explain why Smad6 and Smad7 are tested separately in Figs. 2 and 5. The central question and model should be further probed using single cell RNA-seq approach and/or future experiments to expand and independently validate the present model should be outlined in the Discussion.
Minor points:
1) Abstract: Delete references. Add, if possible model organism studied.
2) Introduction: Add a brief description of Turing networks.
3) Introduction: Early chick lens development is also reviewed by Gunhaga, 2011.
4) Introduction: Additional information on BMP signaling, role of Smad6/7, Fst, and etc. is needed. Explain results of an earlier paper by Grocott et al. in terms of the molecular mechanisms and the present study [18].
Edit Results when this information is presented for the first time.
5) Results: What is the level of Fst expression in the surface ectoderm (Fig. 1f)?
6) Results (p4): In the eye, migratory neural crest is also called periocular mesenchyme.
7) Discussion: Mutual repression of Pax6 and Pax2 is also relevant to the early retinal development (PMID:
11003833).
8) References are not in Development format.

© 2020. Published by The Company of Biologists under the terms of the Creative Commons Attribution License (https://creativecommons.org/licenses/by/4.0/).

First revision

Author response to reviewers' comments

#####################
The following is a point-by-point response indicating how we have addressed the concerns raised (either experimentally or by changes to the text/figures).
#####################

Reviewer 1 Advance Summary and Potential Significance to Field:
Grocott et al propose that a Turing-like network, comprising Pax6/Fst/Tgfb2, is involved in polarising the optic vesicle along its proximal-distal axis. They suggest that in vivo this network cooperates with external signals like BMP to polarise the tissue, but that this network also allows polarity to self-organize as had been observed in retinal organoids. If convincingly shown, I would regard this hypothesis as highly significant and of broad interest to the developmental biology community.

Many aspects of this proposal are well supported by the data provided. In particular, the authors show that interactions between Pax6, Fst and Tgfb2 are consistent with the logic required for a Turing system (summarized in Fig. 4d), and very nicely use simulations to argue that this putative Turing network is sufficient to explain optic vesicle polarization in silico.

Whilst the evidence for each of the interactions in Fig. 4d is good, I think that further evidence is required to show that this network is responsible (necessary? sufficient?) for optic vesicle polarization in vivo or in vitro. In other words, I am convinced that this network exists, but given the data presented, remain unconvinced that it is the primary driver of self-organization.

#####################
As discussed below, we now demonstrate *in vitro* that Tgfb-mediated self-organisation is necessary and sufficient to polarise Pax6 expression.
#####################

Reviewer 1 Comments for the Author:
MAJOR COMMENTS

1. FURTHER EVIDENCE FOR THE ROLE OF PAX6/FST/TGFB2 NETWORK IN OPTIC VESICLE POLARIZATION

I think there are a number of perturbation experiments that could more directly support the hypothesis that the role of the Pax6/Fst/Tgfb2 network is to spontaneously polarize the optic vesicle. However, one would want to see a change in the patterning (e.g. via pax6 expression), that is more subtle than either that pax6 is downregulated (e.g. Fig 5e) or pax6 is unaffected (e.g. Fig 5i).

One possibility I could imagine would be to perform some mosaic perturbation (similar to Fig 5), and show that this can change pax6 patterning throughout the optic vesicle. It would be important to see non-cell-autonomous changes. For example, could the authors use mosaic FstMO or mosaic Fst overexpression (data they may already have) to ask whether they can shift the location of the pax6 domain? The results of these experiments could then be compared to simulations in which the perturbation is applied in silico.

The experimental design in Fig 5g,h could also provide complementary evidence, if I understand it correctly. Here, endogeneous Fst is blocked by the morpholino, but constitutive (i.e. pax6-independent) Fst is provided. This is a nice experiment because it doesn't completely remove Fst, but only removes its transcriptional regulation (including via pax6), which (my intuition suggests) will be important for the self-organizing abilities of the network. From the data presented (Fig 5g), it appears that the vesicle polarises fine – is this to be expected from the simulations?

#####################

© 2020. Published by The Company of Biologists under the terms of the Creative Commons Attribution License (https://creativecommons.org/licenses/by/4.0/).

A major theme of our revised manuscript is that, *in vivo*, there is a wealth of confounding positional information (e.g. extrinsic BMPs, Wnts, Tgfbs, and intrinsic Shh) that heavily constrains the *Pax6*/*Fst*/*Tgfb2* network. Thus, *in vivo* demonstration of self-organisation is experimentally intractable due to this multitude of uncontrollable variables. In other words, *in vivo* data of the kind presented in our previous Fig. 5 (now called Fig. 6) cannot therefore demonstrate self-organisation as the network is not free to express this behaviour.

We therefore demonstrate self-organisation *in vitro* using cultured optic vesicle explants. These experiments are far more tractable, since extrinsic positional information is discarded during dissection and only an intrinsic Shh gradient remains, which we eliminate by pharmacological inhibition.

Fig. 4 has been modified to introduce new 2-D explant simulations and corresponding experiments, as described in lines 188-204 of page 7 of the revised manuscript.

A new Fig. 5 presents a series of 2-D explant simulations and corresponding explant experiments demonstrating that Tgfb-mediated self-organisation is necessary and sufficient to polarise Pax6 in the absence of positional information. These data are described in lines 206-250 starting on page 7 of the revised manuscript. The additional models (Model C & D) have been added to the Supplementary Information and will be made available online via the interactive Jupyter Notebook hosted on GitHub.

We thank the reviewer for requesting further evidence to support this point and trust that these new data make a stronger case for self-organisation.
####################

2. BETTER DESCRIPTION OF TISSUE GEOMETRY

Throughout the manuscript, I was not always clear on the geometry/orientation of the data presented – both experimental and simulations. I would recommend:

a) early on, provide a brief description and schematic of the 3D structure of the optic vesicle, the proximal-distal patterning events and their relation to the vesicle-to-cup transition. Explain how this fits in with the orientation of the in situs presented. (Also: it appears that the morphology varies between different sections, why is this?)

####################
We apologise that tissue geometry was not always clear and accept the reviewer's recommendations.

A new panel A in Fig. 1 shows 3-D reconstructions of optic vesicles/cups at different stages of development, and the horizontal plane of sectioning is indicated.

A new panel D in Fig. 1 shows a labelled 2-D schematic of a horizontal section through the optic vesicle, corresponding to the plane of sectioning shown in Fig. 1A. A full description is provided in the updated figure legend. Please see lines 693-696 on page 22 of the revised manuscript.

Both new panels are cited from the main text.

We have also added new text explaining that "Differences in morphology of sections are due to i) slight differences in staging of embryos between HH10- and HH10+, and ii) slight obliqueness and variation in the dorsal-ventral level of the horizontal sections." Please see lines 492-494 on page 17.
####################

b) When describing the simulations, can you include a longer discussion in the main text on tissue geometry and boundary conditions. In the supplementary movies, it looks like there are 1D domains with periodic boundary conditions. Can the authors justify this choice (or at least discuss it in more detail, particularly from a biological standpoint)?

© 2020. Published by The Company of Biologists under the terms of the Creative Commons Attribution License (https://creativecommons.org/licenses/by/4.0/).

#####################
We apologise for not explaining tissue geometry and boundary conditions in the main text.

For 1-D simulations, we now explain that the single spatial dimension was intended to "represent the optic vesicle's anterior-posterior axis (comprising anterior-proximal, distal and posterior-proximal domains). Simulations were performed with both zero-flux (Fig. 5) and periodic (Supplementary Movies 1 & 2) boundary conditions to represent dissected optic vesicle explants and spherical organoids, respectively." Please see lines 154-157 on page 6 of the revised manuscript.

We have improved Fig. 4 to better explain correspondence between 1-D simulations and tissue geometry.

In Fig. 4 B-B' and D-D' (previously Fig 4 b-c, e-f) the vertical (1-D spatial) axes are now labelled as 'Anterior-Posterior Axis' within which different zones are labelled as either 'Proximal' or 'Distal'.

The following text was added to the legend for Fig. 4: "The vertical y-axis represents the hemispherical optic vesicle's anterior-posterior axis, which is divided into anterior-proximal, distal and posterior-proximal domains". Please see lines 754-756 on page 24 of the revised manuscript.

For 2-D simulations, we now explain that "we explored both zero-flux and fixed boundary conditions, disregarding the latter as the former agreed more closely with experimental observations. It may be interpreted that adsorption of morphogens to extracellular matrix and cell surface proteins within explants prevents a significant outward flux, while the absence of morphogens from the defined bathing medium prevents an inward flux." Please see lines 190-194 on page 7 of the revised manuscript.
#####################

MINOR POINTS
1. Can you give more details on the Fst transgene (as used in Fig 5g,h)?

#####################
We now explain that the Fst transgene encodes the Fst 315 isoform and illustrate this schematically in Fig. 6A. Please see line 273 on page 10 of the revised manuscript.

We also explain that FstMO knocks down both Fst 300 and Fst 315 isoforms. Please see lines 263-264 on page 9 of the revised manuscript.

The following text was added to the legend for Fig. 6 (previously Fig. 5): "Schematic showing domain structures encoded by naturally occurring Fst transcripts. The shorter Fst 300 is generated by alternative splicing. SP, 28 aa signal peptide cleaved co-translationally; NTD, N-terminal domain; FSD, Follistatin domain; AT, acidic tail." Please see lines 798-801 on page 26 of the revised manuscript.
#####################

2.Lines 15-16, page 11: it is unclear a priori that log-transforming guarantees normality for fold changes - you have to make further assumptions (or show/state that log transforming makes your data closer to normally distributed).

#####################
We apologise for not reporting that Shapiro-Wilk normality tests were performed to verify that log-transforming our fold-change data brings it closer to normally distributed.

We now report that fold-change data was "log-transformed to bring data closer to a normal distribution (verified by Shapiro-Wilk test) prior to plotting and null hypothesis significance testing." Please see lines 456-457 on page 16 and lines 508-510 on page 18 of the revised manuscript.
#####################

3. Fig 4k-m are not like-for-like comparisons. In the simulations, patterns are presented; whereas in the experimental data, overall levels of expression are presented. It is hard to

© 2020. Published by The Company of Biologists under the terms of the Creative Commons Attribution License (https://creativecommons.org/licenses/by/4.0/).

compare the two. Can the patterning of the tissues in Fig 4m be assayed? If not, can the simulations be used to generate an in silico version of Fig 4m?

#####################
We apologise for this discrepancy and are very grateful to the reviewer for raising this point as it led us to a tractable *in vitro* assay for self-organisation as described above.

The 1-D simulation and RT-QPCR data from the previous Fig. 4k-l & m has been replaced in favour of simulations and experiments that more directly addresses *Pax6* patterning in cultured optic vesicle explants. To better simulate Pax6 patterning in explant experiments, we developed a 2-D explant model (Model C), which is fully described in the expanded Supplementary Information. Please see lines 187-204 on page 7 of the revised manuscript.

New Fig. 4 panels H-H' include 2-D explant simulation data and panels I-J' include new explant experimental data suggesting that the Pax6+ distal pole dynamically repolarises during explant culture.
#####################

4. In the supplementary movies (and in Fig 4e), brief oscillations in Pax6 are seen - is this seen in vivo/in vitro? Is this a by-product of the initial conditions chosen?

#####################
We have now included the following paragraph regarding oscillations in the Discussion section:

"In some of our simulations the Pax6/Fst/Tgfb2 network is observed to oscillate (Fig. 4D-D'; Supplementary Movie 2). The potential for oscillation derives from the Eigenvalues associated with the Turing condition and thus from the model's governing equations and parameter choices. For example, in Model B this tendency to oscillate may be suppressed by increasing the negative feedback that Tgfb2 exerts on Pax6. Whether or not oscillations manifest in a given simulation is further influenced by the choice of initial conditions. For example, Model B is observed to oscillate during de novo pattern formation (Fig. 4D-D'; Supplementary Movie 2), but not when elaborating an existing pre-pattern (equivalent to the Model A simulation in Fig. 4B-B'). For this reason, we might expect that oscillations are more likely to arise during de novo pattern formation in retinal organoid cultures and less so in the embryo where a wealth of positional information constrains the Pax6/Fst/Tgfb2 network. Whether or not this gene network oscillates in vitro or in vivo is yet to be investigated.

Please see lines 375-387 on page 13 of the revised manuscript.
#####################

5.When discussing the data from Fig 4g-m, it should be emphasized that these predictions (i.e. larger domain gives multiple poles, smaller domain can result in no patterning) is a rather general feature of Turing systems and not a specific test of the Pax6/Fst/Tgfb2 network being responsible.

#####################
We thank the reviewer for raising this point.

As described above, the previous Fig. 4 g-m have been replaced by new 2-D simulated and experimental explant data.

However, we have modified the text introducing these new experiments to emphasize the generality of this feature: "Similarly, reducing tissue size limits the number rather than the size of pattern elements generated by a Turing network so that for example, a single 'spot', half a 'spot' (i.e. a gradient) or no 'spot' is generated. " Please see lines 183-185 on page 7 of the revised manuscript.
#####################

***** Reviewer 2 Advance Summary and Potential Significance to Field:
The manuscript by Timothy Grocott and co-workers tackles a key question of developmental

© 2020. Published by The Company of Biologists under the terms of the Creative Commons Attribution License (https://creativecommons.org/licenses/by/4.0/).

biology, the pattern formation in early vertebrate embryos. They use patterning of early optic chick vesicle as an experimental model. Their central hypothesis is that a lineage-specific DNA-binding transcription factor Pax6 is a cornerstone of a cross-talk of multiple signaling pathways and these are wired together as a putative Turing network. The experiments test this model through three components of TGF/BMP signaling, Bmp4, follistatin (Fst), and Tfgb2 at their mRNA levels. The main findings in the paper are summarized in a model that accounts for differences of Pax6 regulation in distal and proximal regions of the optic vesicle (Fig. 6). Overall, these findings are novel and within the scope of journal Development.

Reviewer 2 Comments for the Author:
There is no direct relationship established between the Pax6 and Vsx2 expression in the retina. Additional
genes, such as Fgf9 and Erk1/2, should be examined.

#####################
We thank the reviewer for this observation. Although we do not state that *Vsx2* is a direct target of *Pax6*, we accept that we could be much clearer on this point.

The interaction between *Pax6* and *Vsx2*, and those between *Tgfb2* and *Mitf/Wnt2b* are now indicated with broken lines in Fig. 7A-B (previously Fig. 6a-b). The legend for Fig. 7 now includes the text "Interactions indicated by broken lines may be indirect". Please see line 834 on page 27 of the revised manuscript.
#####################

Auto-regulation of Pax6 in the optic vesicle was probed through dnPax6 mis-expression. This experiment requires additional data to determine levels of ectopic expression in relationship to their endogenous levels.

#####################
We apologise for not including this control. We now provide additional evidence in Fig. 2G-G' showing the extent of exogenous *dnPax6* expression relative to endogenous *Pax6* expression.

The main text now includes the following: "To confirm that *dnPax6* was overexpressed relative to endogenous *Pax6*, an N-terminal riboprobe was used to collectively detect both endogenous *Pax6* and exogenous *dnPax6* expression (Fig. 2G)". Please see lines 117-119 on page 4 of the revised manuscript.

The legend for Fig. 2 now includes the following: "G) Endogenous Pax6 and exogenous dnPax6 gene expression following transfection with dnPax6 + GFP, and G') anti-GFP immuno showing location of dnPax6 + GFP transfected cells. Note that immunofluorescence in G' is heavily quenched by strong in situ staining." Please see lines 724-727 on page 23 of the revised manuscript.
#####################

Explain why Smad6 and Smad7 are tested separately in Figs. 2 and 5.

#####################
Smad6 and Smad7 are tested separately because they act in different pathways; Bmp and Tgfb/Activin/Nodal pathways, respectively.

We apologise if this was unclear and now explain this point with a paragraph outlining Tgfb-superfamily signalling in the introduction. Please see lines 48-57 on page 2 of the revised manuscript.
#####################

The central question and model should be further probed using single cell RNA-seq approach…

#####################
While we thank the reviewer for this suggestion, we agree with the editor that scRNA-Seq is beyond the scope of the current study.

© 2020. Published by The Company of Biologists under the terms of the Creative Commons Attribution License (https://creativecommons.org/licenses/by/4.0/).

Since scRNA-Seq obliterates the spatial relationships between cells, it is not obvious that this approach would address our central question regarding self-organisation of spatial patterns. In any case, we would be unable to perform these experiments within the normal timeframe for revision.
#####################

…and/or future experiments to expand and independently validate the present model should be outlined in the
Discussion.

#####################
We thank the reviewer for this suggestion and include a “Future Directions” section within the discussion to outline future experiments. Please see lines 389-408 on page 14 of the revised manuscript.
#####################

Minor points:
1)Abstract: Delete references. Add, if possible model organism studied.

#####################
We have removed references from the Abstract and highlight use of chick as a model organism. Please see line 14 on page 1 of the revised manuscript.
#####################

2)Introduction: Add a brief description of Turing networks.

#####################
We apologise for this omission.

A brief description of Activator-Inhibitor type Turing networks in now included in the introduction and supported by new Fig. 1 panels B & C. Please see lines 62-70 on page 3 of the revised manuscript.
#####################

3)Introduction: Early chick lens development is also reviewed by Gunhaga, 2011.

#####################
We apologise for this omission.

Gunhaga, 2011 is now cited in the introduction. Please see line 34 on page 2 of the revised manuscript.
#####################

4)Introduction: Additional information on BMP signaling, role of Smad6/7, Fst, and etc. is needed. Explain results of an earlier paper by Grocott et al. in terms of the molecular mechanisms and the present study [18].
Edit Results when this information is presented for the first time.

#####################
The introduction now includes a paragraph outlining Tgfb-superfamily (Tgfb/Activin/Nodal and BMP) signalling and introducing the earlier result of Grocott et al 2007. Please see lines 48-61 starting on page 2 of the revised manuscript.
#####################

5) Results: What is the level of Fst expression in the surface ectoderm (Fig. 1f)?

#####################
We did not detect any *Fst* expression in the surface ectoderm in Fig. 1J (previously Fig. 1f). We now note the absence of *Fst* and *Tgfb2* expression in the surface ectoderm from the main text. Please see lines 84-85 on page 3.
#####################

© 2020. Published by The Company of Biologists under the terms of the Creative Commons Attribution License (https://creativecommons.org/licenses/by/4.0/).

6) Results (p4): In the eye, migratory neural crest is also called periocular mesenchyme.

#####################
We now note that neural crest cells contribute to the peri-ocular mesenchyme. Please see lines 154-155 on page 5 of the revised manuscript.
#####################

7) Discussion: Mutual repression of Pax6 and Pax2 is also relevant to the early retinal development (PMID: 11003833).

#####################
This point directly relates to the role of intrinsic Shh positional information within the optic vesicle, which we now address experimentally as described above.

The discussion now includes the following paragraph highlighting mutual repression of Pax6 and Pax2:

“In Model D we accounted for intrinsic positional information by incorporating direct suppression of Pax6 expression by a ventral-high to dorsal-low gradient of Shh activity (Fig. 5; Supplementary Information) (Ekker et al., 1995; Macdonald et al., 1995). This is a convenient abstraction however; at later stages, the ventral extent of Pax6 expression in vivo is refined via reciprocal inhibition between distal Pax6 (prospective neural retina) and ventral Pax2 (prospective optic stalk) (Schwarz et al., 2000), whose own expression is activated by ventral Shh (Ekker et al., 1995; Macdonald et al., 1995).”

Please see lines 364-370 on page 12 of the revised manuscript.
#####################

8) References are not in Development format.

#####################
The references have been re-formatted in the style of Development.

Other changes to the manuscript:

We have taken the opportunity to add sub-headings to the Results and Discussion sections.

The model equations have been improved to prevent divide-by-zero errors arising during simulations. These are fully described in Supplementary Information. This was a necessary change to permit 2-D simulations on arbitrary shaped domains (i.e. simulation of explant experiments, where pixels outside of the explant domain have zero concentration values). The simulations in Fig. 4 were repeated using the revised equations and were found to give qualitatively identical results.

Simulation data and Pax6 immunofluorescence data in Fig. 4 & 5 are now rendered using a perceptually uniform colour-map (mpl-inferno) that is robust to colour-blindness.
#####################

Second decision letter

MS ID#: DEVELOP/2019/185827

MS TITLE: The Pax6 master control gene initiates spontaneous retinal development via a self-organising Turing network

AUTHORS: Timothy Grocott, Estefania Lozano Velasco, Gi Fay Mok, and Andrea E Munsterberg

© 2020. Published by The Company of Biologists under the terms of the Creative Commons Attribution License (https://creativecommons.org/licenses/by/4.0/).

I have now received all the referees reports on the above manuscript, and have reached a decision. The referees' comments are appended below, or you can access them online: please go to BenchPress and click on the 'Manuscripts with Decisions' queue in the Author Area.

The overall evaluation is positive and we would like to publish a revised manuscript in Development, provided that the referees' comments can be satisfactorily addressed. Referee 1 requests that you include the data showing that changing the direction of the Shh gradient reverses the polarity of Pax6 expression. I agree that adding these data will help illustrate the model and strengthen the argument. Please attend to all of the reviewers' comments in your revised manuscript and detail them in your point-by-point response. If you do not agree with any of their criticisms or suggestions explain clearly why this is so.

We are aware that you may currently be unable to access the lab to undertake experimental revisions. If it would be helpful, we encourage you to contact us to discuss your revision in greater detail. Please send us a point-by-point response indicating where you are able to address concerns raised (either experimentally or by changes to the text) and where you will not be able to do so within the normal timeframe of a revision. We will then provide further guidance. Please also note that we are happy to extend revision timeframes as necessary.

Reviewer 1

Advance summary and potential significance to field

Grocott et al propose that a Turing-like network, comprising Pax6/Fst/Tgfb2, is involved in polarising the optic vesicle along its proximal-distal axis. Their revisions have satisfied the concerns raised in my original review, and I recommend this article for publication.

Comments for the author

OVERALL COMMENTS

The authors added substantial additional data and these significantly improve the manuscript. Showing the re-orientation of the Pax6 pole in explants (and in silico) is a great demonstration of a self-regulating system. The TGFB and HH perturbations further strengthen their TGFB-centric model; Fig 5E,G,I are a highlight. I enjoyed reading the revision!

MINOR COMMENTS
Minor comments made previously have been addressed (the discussions/figs on geometry work well).

Small point: on line 229 it says that data "not shown"; could you include it?

Reviewer 2

Advance summary and potential significance to field

The authors have addressed all major points of criticism and very much improved their manuscript. They examine very important developmental process and compare their findings with self-organizing formation of optic cups from mammalian ES cells. They probe their system through a small set of proteins and clearly demonstrate how their mutual regulation can elicit the patterning events.

Comments for the author

None.

© 2020. Published by The Company of Biologists under the terms of the Creative Commons Attribution License (https://creativecommons.org/licenses/by/4.0/).

Second revision

Author response to reviewers' comments

#####################

We thank both referees for their time and valuable suggestions.

The following is a point-by-point response indicating how we have addressed the concerns raised.

#####################

Reviewer 1 Advance Summary and Potential Significance to Field:
Grocott et al propose that a Turing-like network, comprising Pax6/Fst/Tgfb2, is involved in polarising the optic vesicle along its proximal-distal axis. Their revisions have satisfied the concerns raised in my original review, and I recommend this article for publication.

Reviewer 1 Comments for the Author:
OVERALL COMMENTS

The authors added substantial additional data and these significantly improve the manuscript. Showing the re-orientation of the Pax6 pole in explants (and in silico) is a great demonstration of a self-regulating system. The TGFB and HH perturbations further strengthen their TGFB-centric model; Fig 5E,G,I are a highlight. I enjoyed reading the revision!

MINOR COMMENTS

Minor comments made previously have been addressed (the discussions/figs on geometry work well).

Small point: on line 229 it says that data "not shown"; could you include it?

#####################

We have now included this simulation data as new panels (D & D') in Fig. 5. The text on line 222 of page 8 of the revised manuscript was modified as follows: "Moreover, inverting the Shh gradient (Fig. 5D) caused a reversal of *Pax6* polarity (Fig. 5D')."

The following text was added to the legend for Fig. 5: "**D-D')** 2-D numerical simulation of Model D showing **D)** reversal of the Shh gradient and **D')** corresponding reversal of Pax6 polarity." Please see lines 782-783 on page 25 of the revised manuscript.

The remaining panels of Fig. 5 have been re-labelled and all references to these have been updated in the main text.

#####################

***** Reviewer 2 Advance Summary and Potential Significance to Field:
The authors have addressed all major points of criticism and very much improved their manuscript. They examine very important developmental process and compare their findings with self-organizing formation of optic cups from mammalian ES cells. They probe their system through a small set of proteins and clearly demonstrate how their mutual regulation can elicit the patterning events.

Reviewer 2 Comments for the Author:
None.

© 2020. Published by The Company of Biologists under the terms of the Creative Commons Attribution License (https://creativecommons.org/licenses/by/4.0/).

Third decision letter

MS ID#: DEVELOP/2019/185827

MS TITLE: The Pax6 master control gene initiates spontaneous retinal development via a self-organising Turing network

AUTHORS: Timothy Grocott, Estefania Lozano Velasco, Gi Fay Mok, and Andrea E Munsterberg
ARTICLE TYPE: Research Article

I am happy to tell you that your manuscript has been accepted for publication in Development, pending our standard ethics checks.

© 2020. Published by The Company of Biologists under the terms of the Creative Commons Attribution License (https://creativecommons.org/licenses/by/4.0/).
